# Supplementary material for: Light intensity-induced photocurrent switching effect
Source: Nat Commun. 2020 Feb 12;11:854. doi: 10.1038/s41467-020-14675-5 (PMC7016128; doi:10.1038/s41467-020-14675-5)
Supplement: Supplementary file 1 — Supplementary Info [file 41467_2020_14675_MOESM1_ESM.pdf]

Supplementary information for

Light Intensity Induced Photocurrent Switching Effect

Agnieszka Podborska[1], Maciej Suchecki[1,2], Krzysztof Mech[1], Mateusz Marzec[1], Kacper Pilarczyk[1*], Konrad Szaciłowski[1]

[1]Academic Centre for Materials and Nanotechnology, AGH University of Science and Technology, al. A. Mickiewicza 30, 30-059 Kraków, Poland.

[2]Faculty of Physics and Applied Computer Science, AGH University of Science and Technology, al. A. Mickiewicza 30, 30-059 Kraków, Poland.

[*]E-mail address: kpilarcz@agh.edu.pl

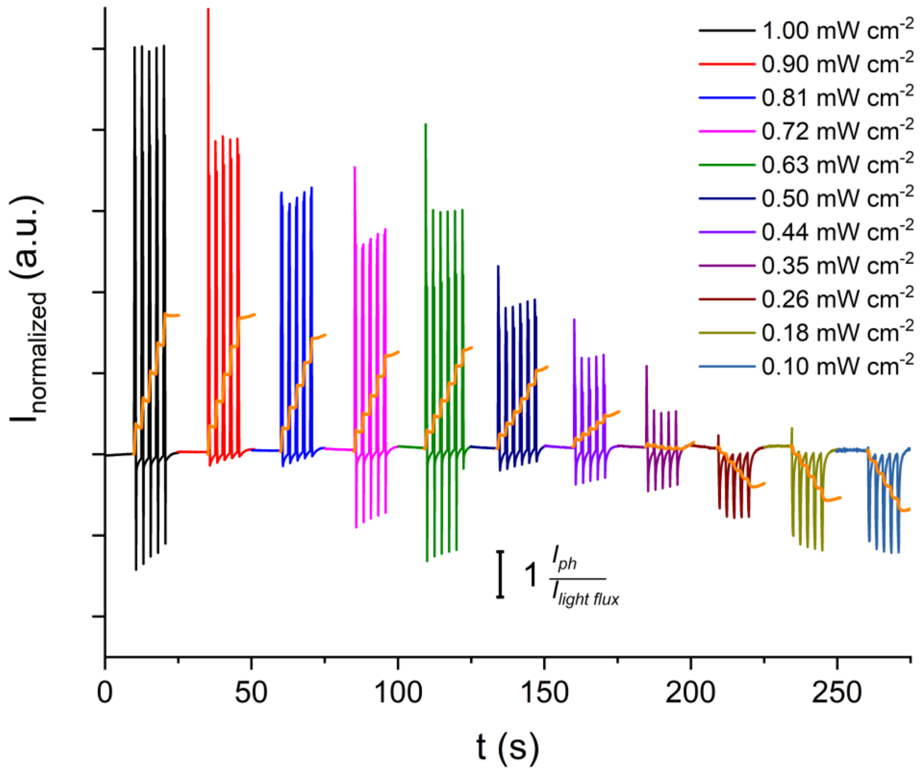

Supplementary Figure 1. The experiment in which Light Intensity Induced Photocurrent Switching (LIIPS) is demonstrated for numerous incident light (λ=365 nm) intensities. The ITO@PET electrode covered with CA-COH@ZnO ternary hybrid material was irradiated at -300 mV vs. Ag/AgCl in oxygen-rich electrolyte. The orange curves represent integrals calculated for each portion of data and their variability may be used to trace how the photocurrent polarity switching is progressing.

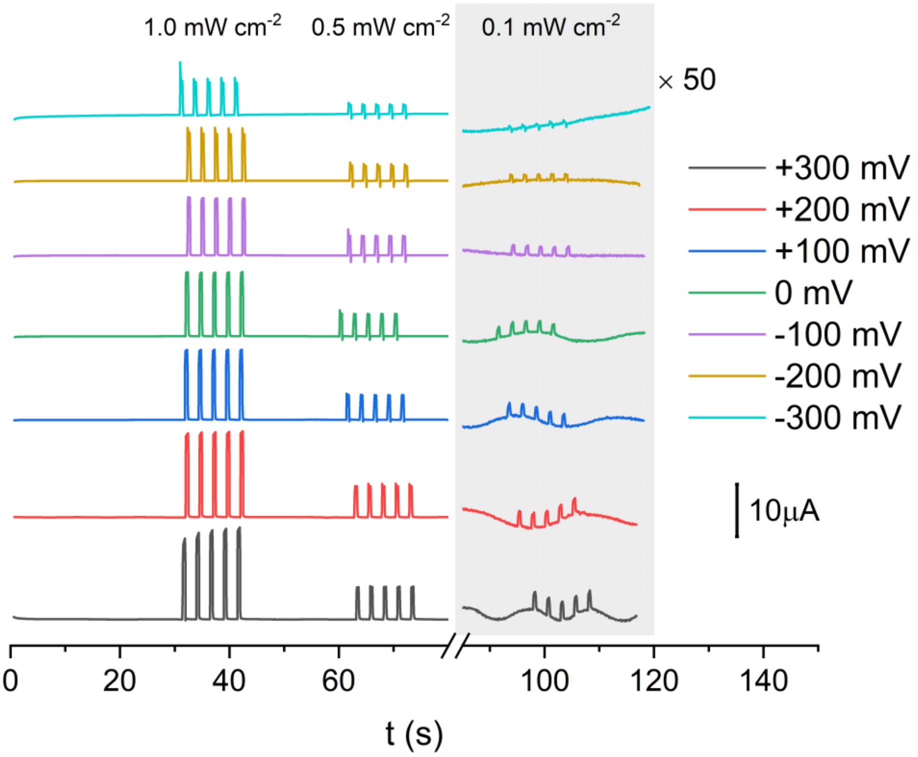

Supplementary Figure 2. The attempt to recreate Light Intensity Induced Photocurrent Switching (LIIPS) effect without the use of light during the conditioning step. The response of ITO@PET electrode covered with CA-COH@ZnO ternary hybrid material was recorded at different potentials applied during the measurement. No photocurrent polarity switching has been observed.

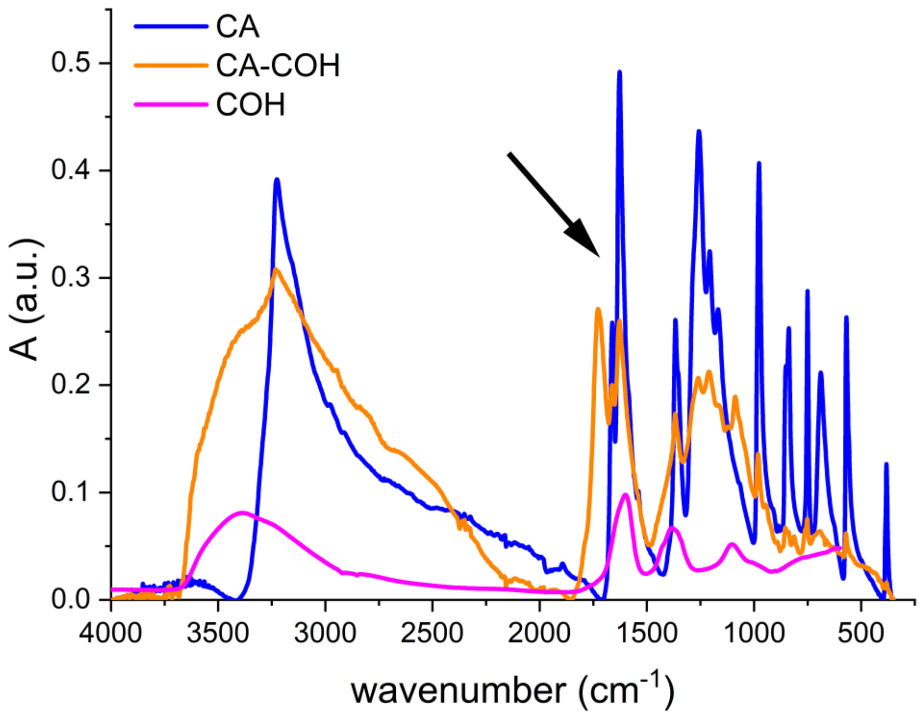

Supplementary Figure 3. The FTIR spectra of modifiers used for the surface modification of zinc oxide. The arrow indicates a shift towards higher wavenumbers of a band which can be associated with C=O stretching mode originating from CA.

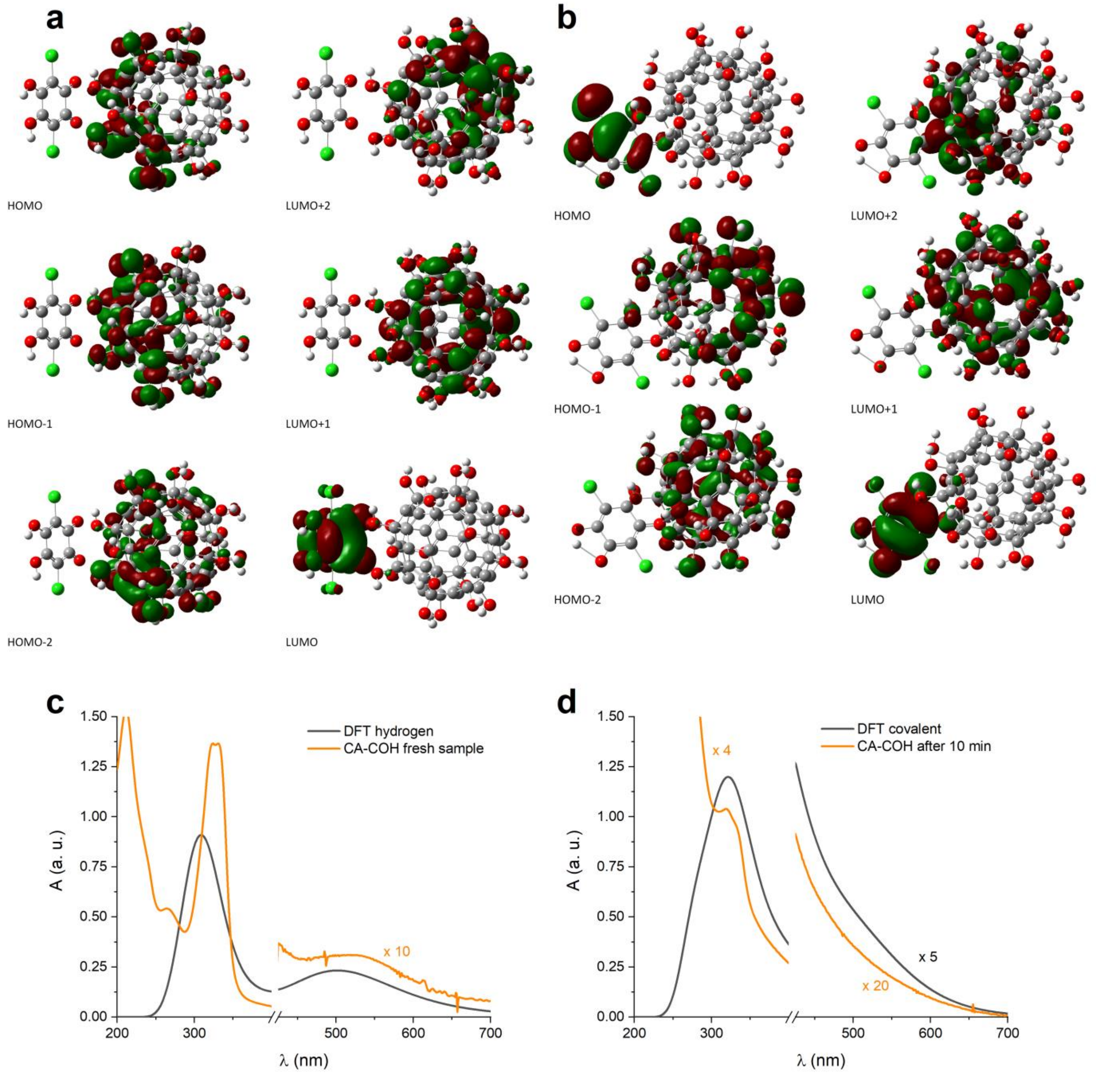

Supplementary Figure 4. The models (with optimized geometry) used in DFT calculations with simulated UV-Vis spectra for CA-COH system. The projections of HOMO and LUMO envelopes for the modifiers coupled through a hydrogen bond (a) or a covalent bond (b) and simulated UV-Vis spectra with an additional band at approximately 550 nm for the former case (c), which vanishes for the latter scenario (d) are shown. All DFT and TD-DFT calculations have been performed at B3LYP/3-21(6D, 7F) level of theory. The electronic absorption spectra have been calculated taking into account first 90 excitations and a peak broadening of 0.333 eV (2685.83 cm^{-1}).

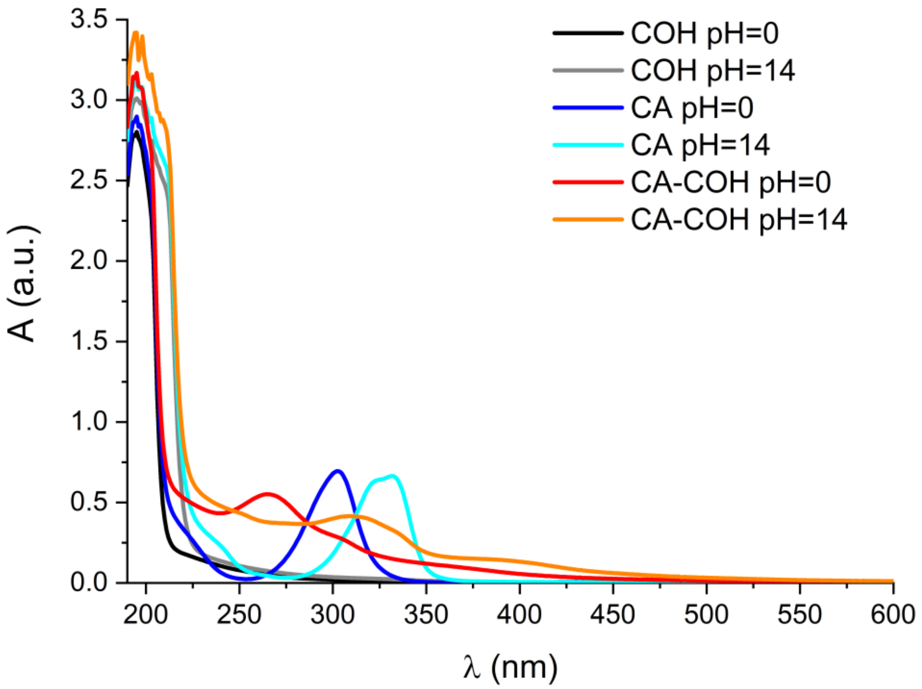

Supplementary Figure 5. The UV-Vis spectra recorded for individual modifiers and their mixture with varying pH of the solution. As expected, the bathochromic shift is observed for individual modifiers and their blend when pH is increased.

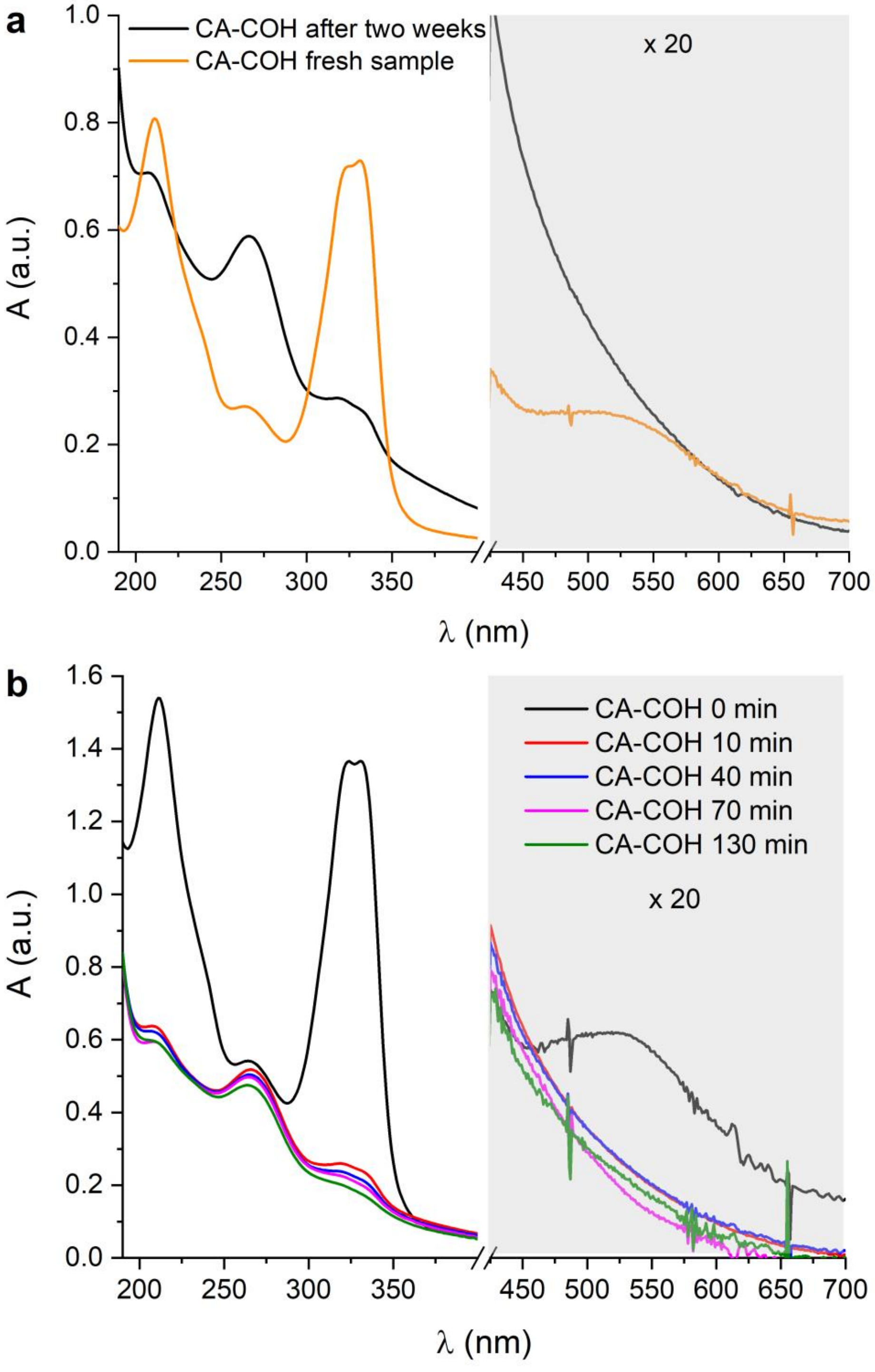

Supplementary Figure 6. The UV-Vis spectra recorded for the mixture of modifiers. The results obtained for a fresh sample and after two weeks (a) and the impact of irradiation on the sample (b) are shown. An additional band at approximately 550 nm can be noticed, which vanishes both after aging and irradiation.

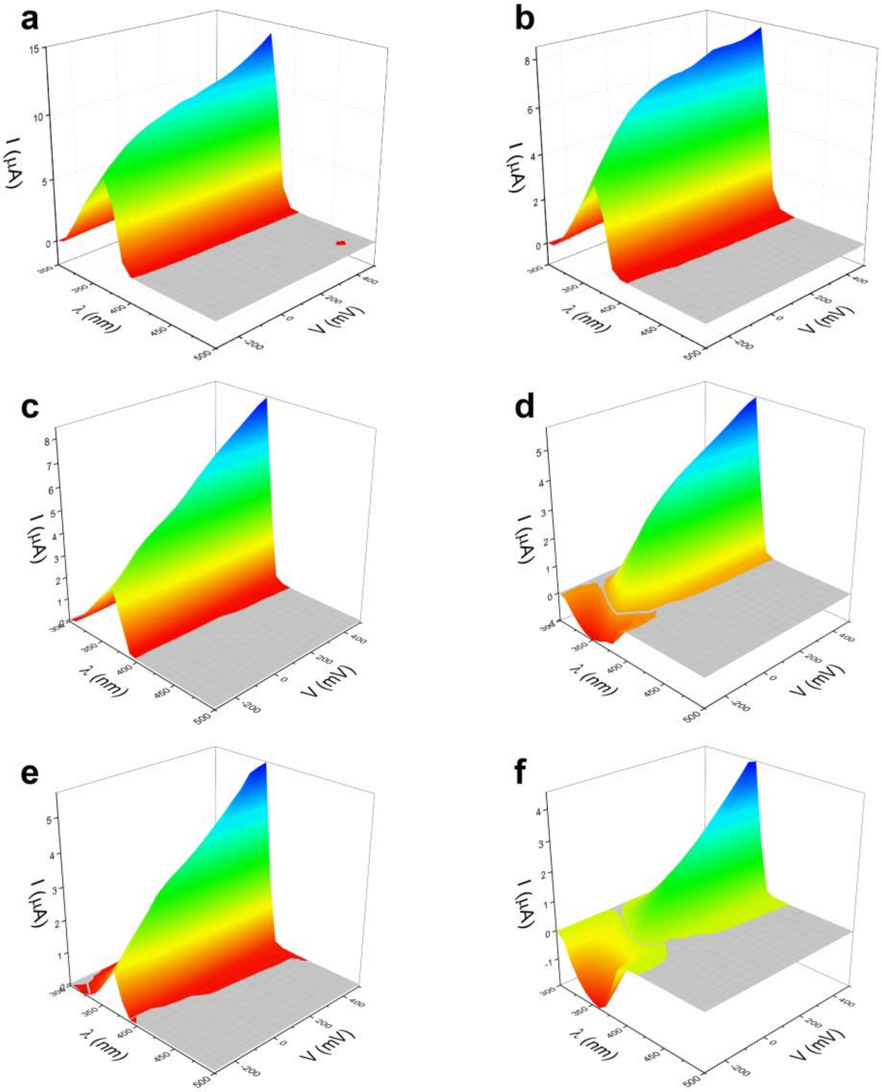

Supplementary Figure 7. The photocurrent action maps recorded for the investigated materials. The measurements were conducted in a three electrode setup with 0.1M KNO_3 as electrolyte purged with argon (left column) or oxygen (right column) for ZnO (a) and (b), CA@ZnO (c) and (d) or CA-COH@ZnO (e) and (f).

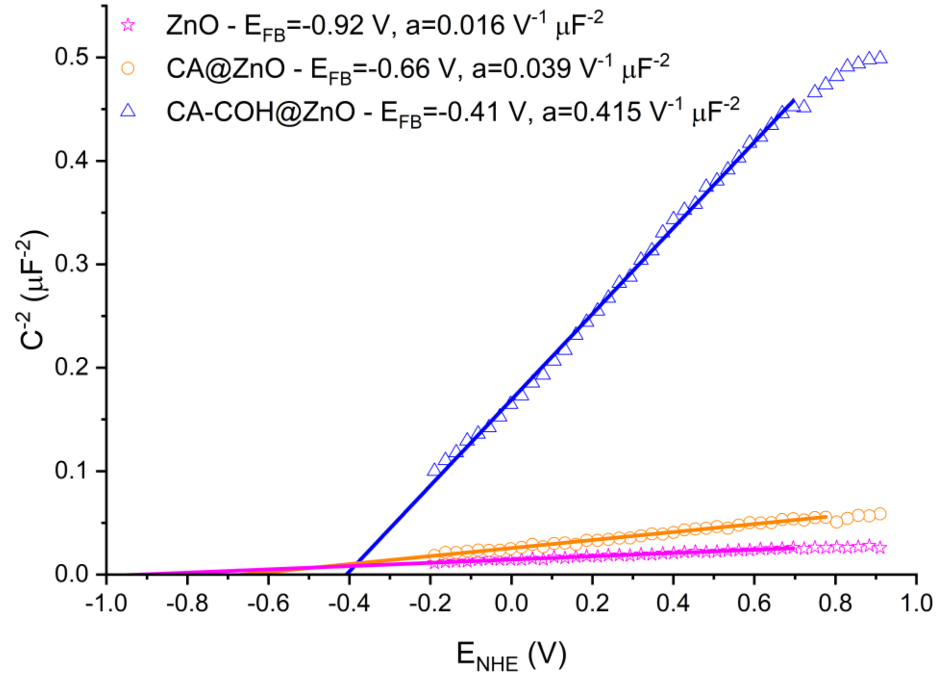

Supplementary Figure 8. The Mott-Schottky plots and estimated flat band potentials for the investigated materials. The measurements were conducted at the frequency of 1 kHz and with the modulation amplitude of 5 mV.

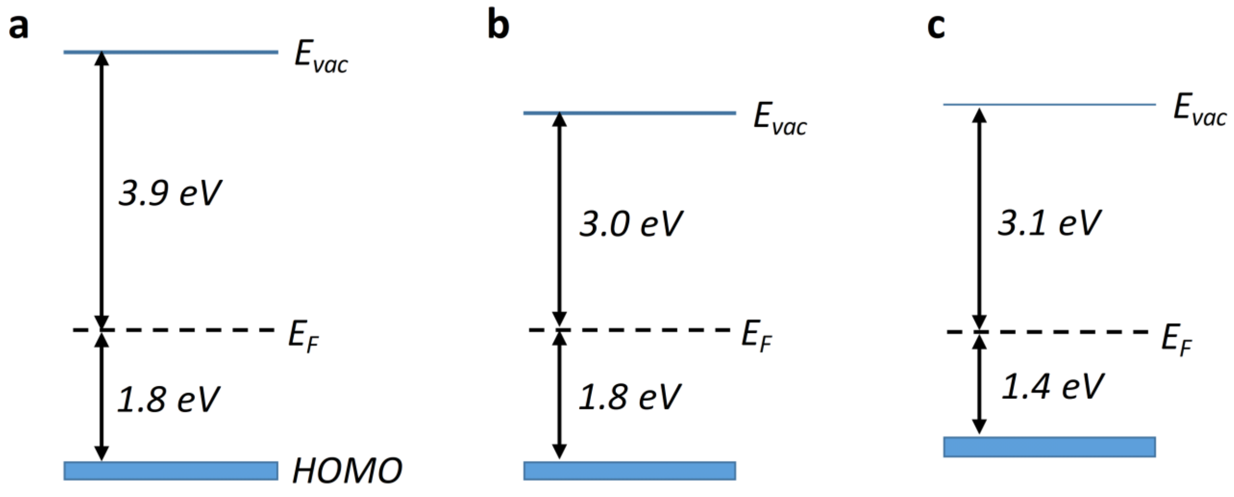

Supplementary Figure 9. The energy diagram constructed based on the UPS measurements. The results for (a) neat zinc oxide, (b) CA@ZnO composite and (c) CA-COH@ZnO ternary hybrid all deposited onto ITO@PET. E_F is the Fermi level, E_{vac} is the vacuum level outside the surface and the HOMO level is also depicted. The distance between E_F and E_{vac} is equal to the work function of a given material and the HOMO to E_F distance is a hole injection barrier.

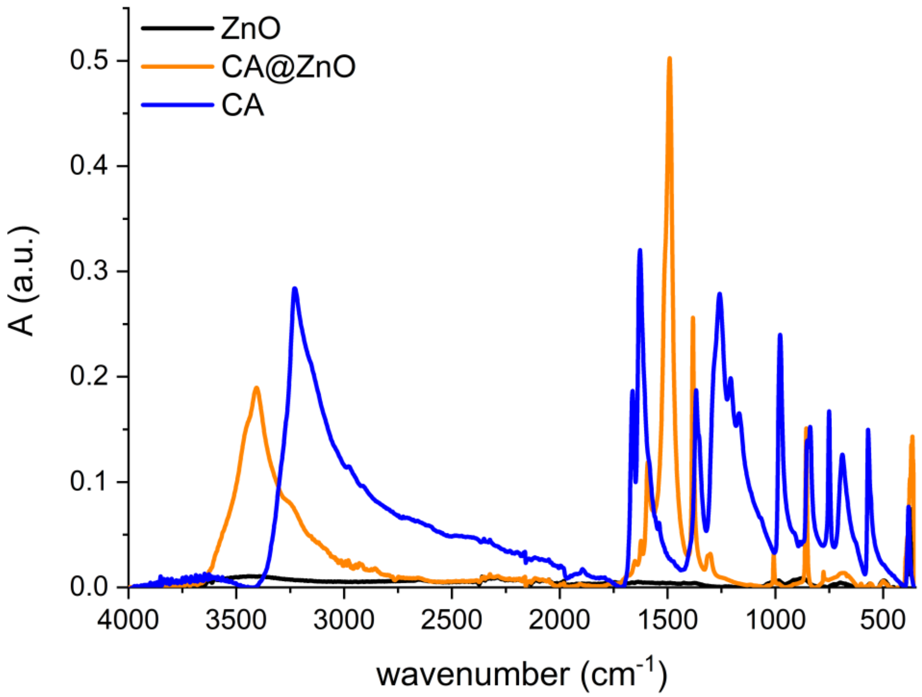

Supplementary Figure 10. The FTIR spectra of neat zinc oxide, chloranilic acid and the hybrid CA@ZnO. Significant shifts and/or partial quenching of absorption bands originating from hydroxyl and carbonyl groups of CA may be noticed.

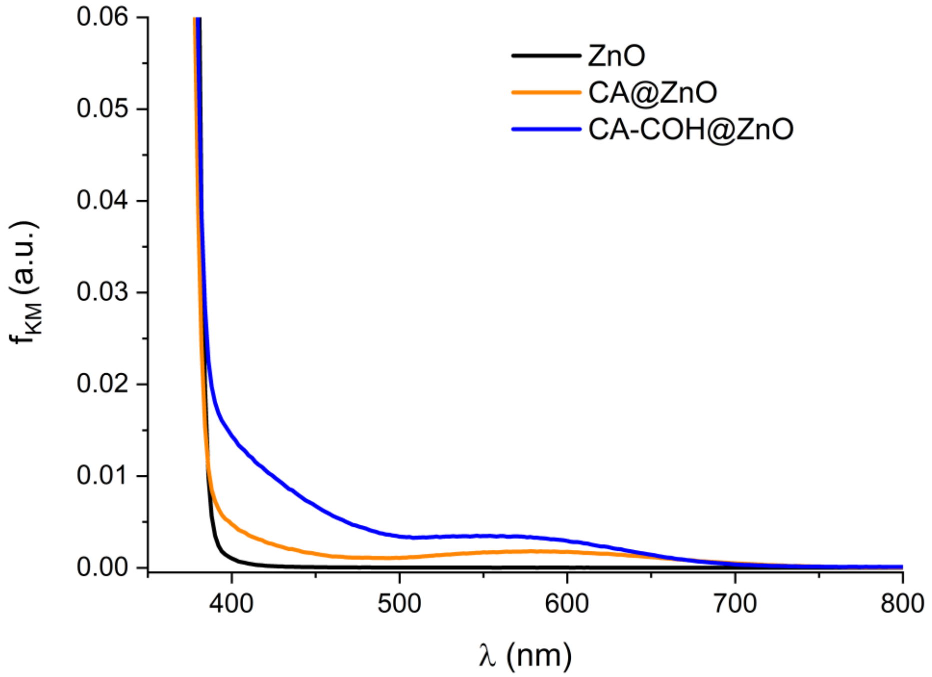

67 **Supplementary Figure 11. The UV-Vis spectra (represented by Kubelka-Munk function) recorded for neat zinc oxide and two hybrid materials.**

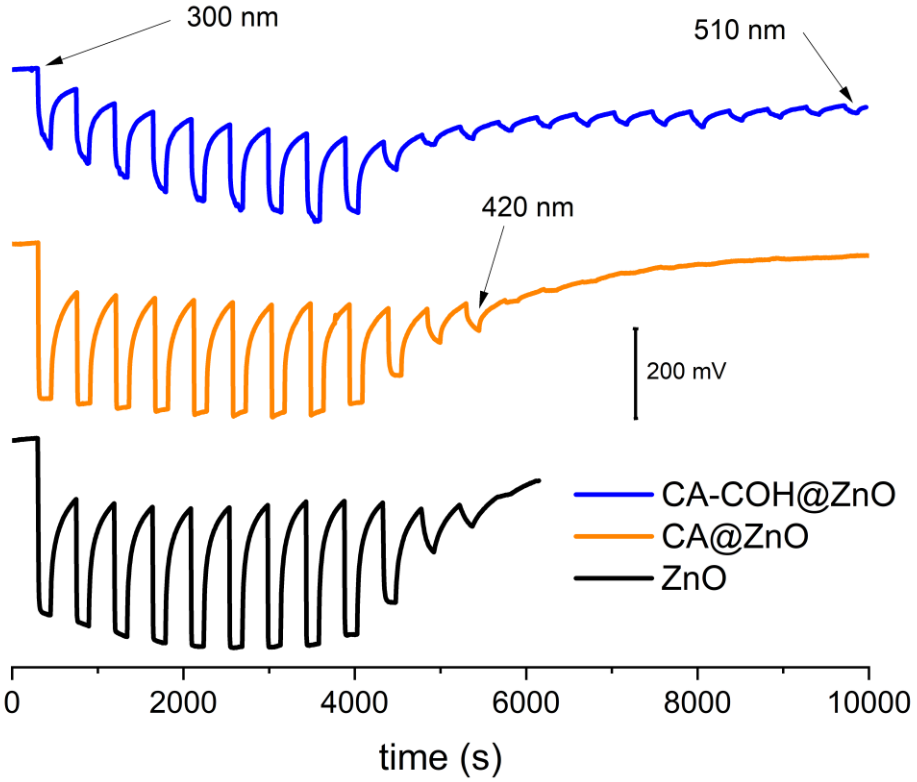

Supplementary Figure 12. The results of surface photovoltage measurements. The spectra were recorded for neat zinc oxide and two hybrid materials with a wavelength step of 10 nm.

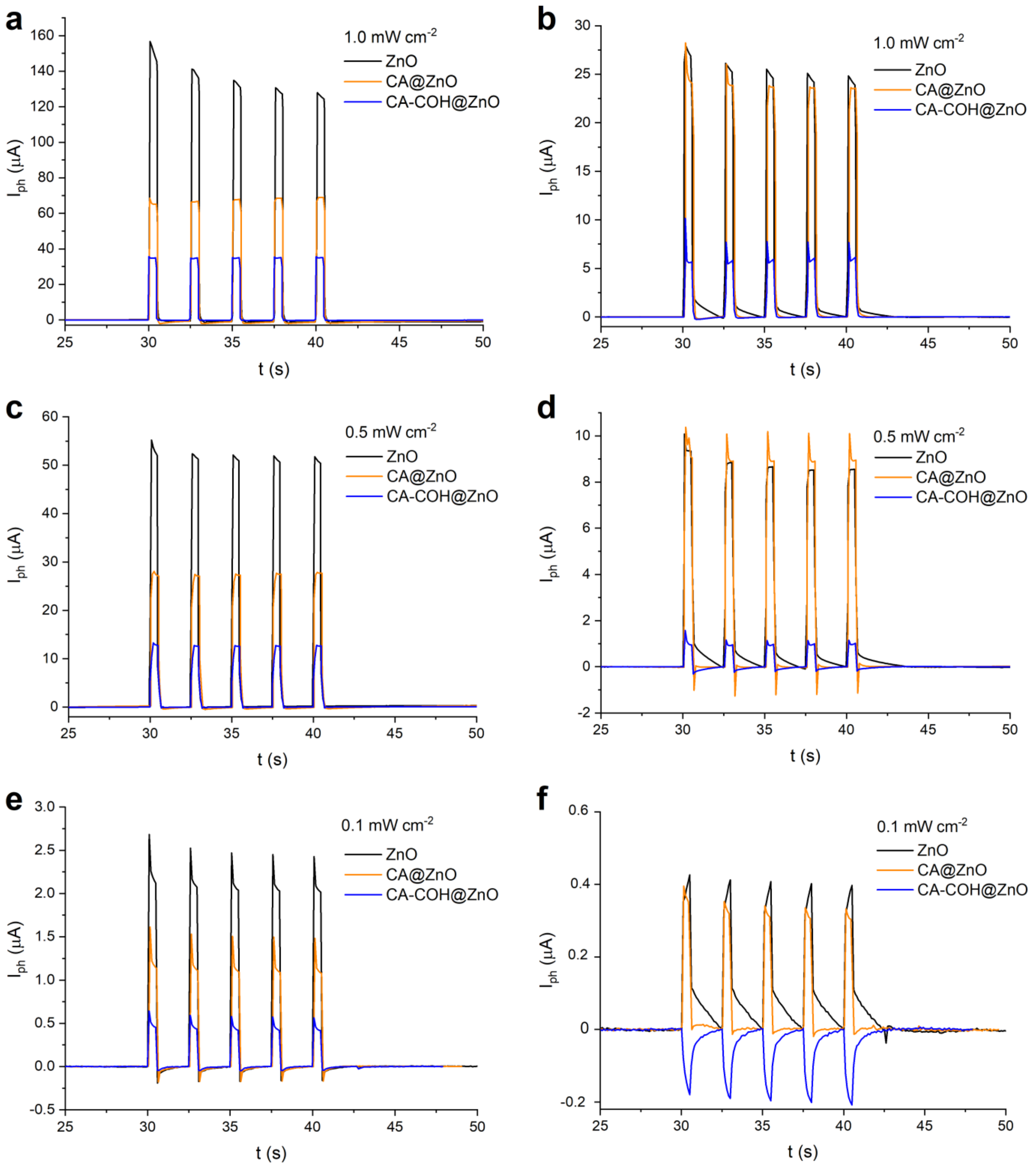

Supplementary Figure 13. The photocurrent profiles recorded for neat zinc oxide and two studied hybrid materials. The electrode potential of -0.3 V was applied in the oxygen-rich conditions with the irradiation wavelength equal to 365 nm and three different light intensities. On the left, the results without the pre-treatment procedure, on the right, the response after conditioning.

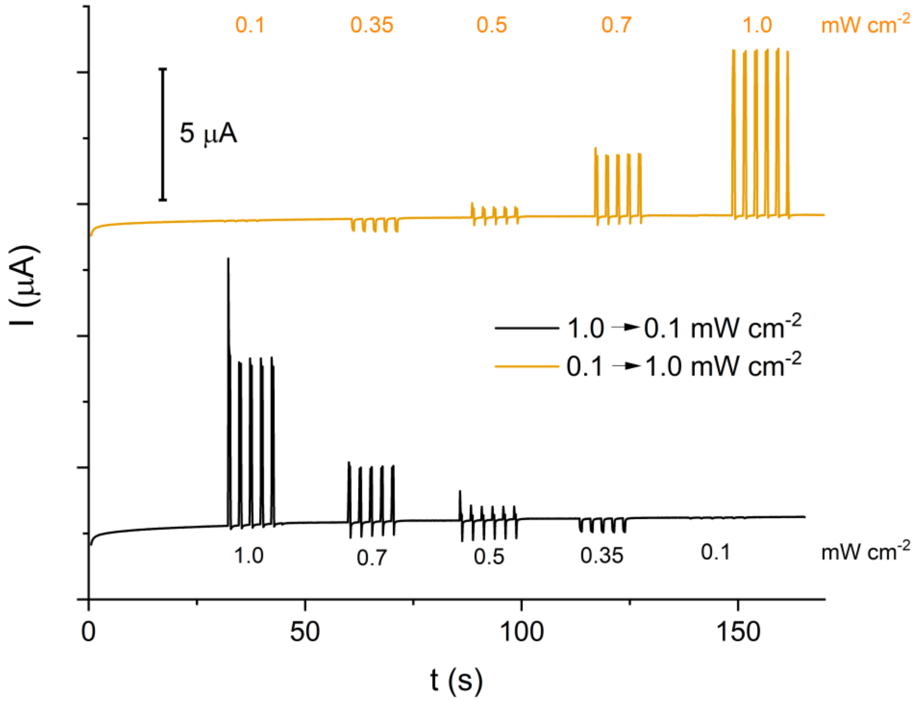

Supplementary Figure 14. The experiment in which the lack of dependency of Light Intensity Induced Photocurrent Switching (LIIPS) on the direction of light (λ=365 nm) intensity changes is demonstrated. The response of ITO@PET electrode covered with CA-COH@ZnO ternary hybrid material at -300 mV vs. Ag/AgCl in oxygen-rich electrolyte was recorded. The labels indicate the light intensity value in mW cm^{-2}.

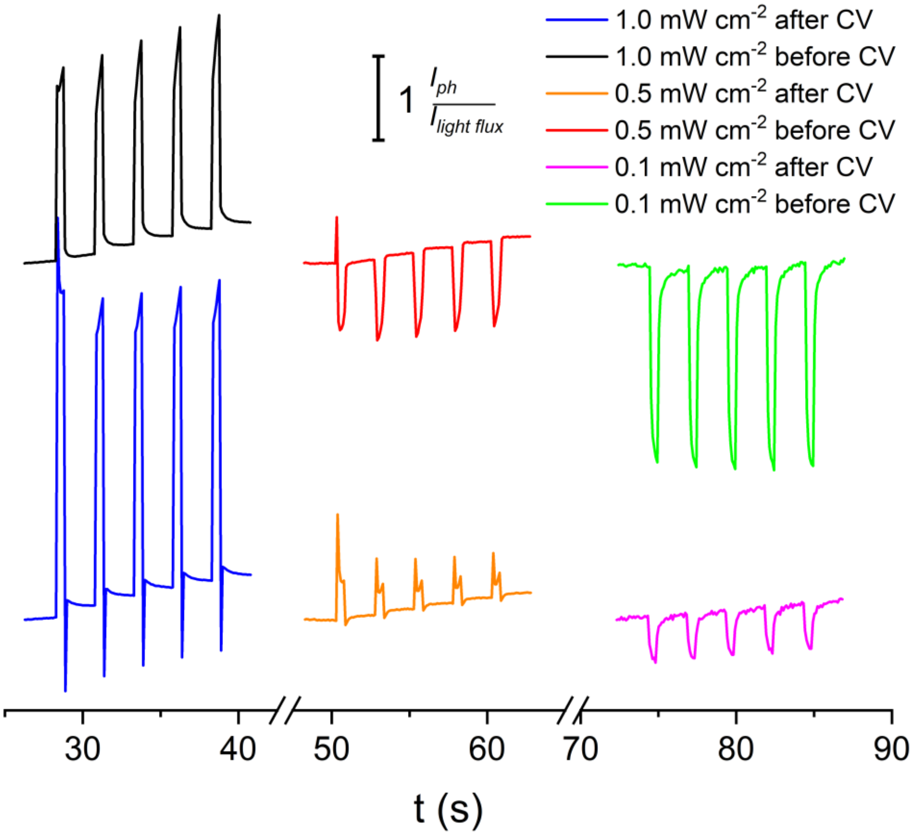

Supplementary Figure 15. The impact of an excessive cyclic voltammetry treatment on Light Intensity Induced Photocurrent Switching (LIIPS). The response of ITO@PET electrode covered with CA-COH@ZnO ternary hybrid material at -300 mV vs. Ag/AgCl irradiated at λ=365 nm in oxygen-rich electrolyte was recorded before and after the cyclic voltammetry treatment (10 scans in the potential range of -0.4÷+0.5 V vs. Ag/AgCl at the scan rate of 50 mV s^{-1}). The significant change in the sample response occurs within the transitional region of light intensities for which the interplay between anodic and cathodic photocurrent generation processes can be easily perturbed.

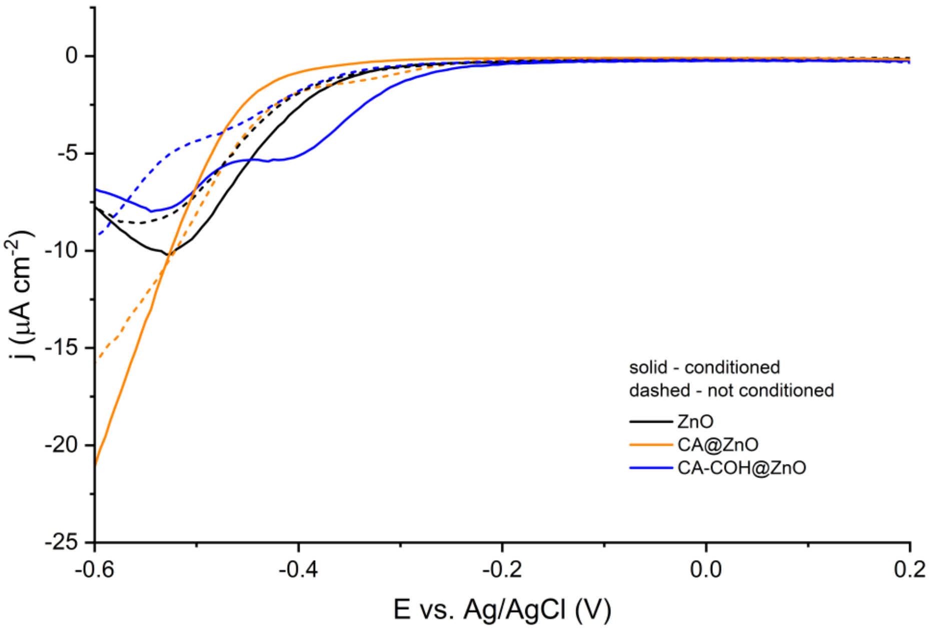

Supplementary Figure 16. The results of DPV measurements for neat zinc oxide and two studied hybrids. Fresh samples (marked with the dashed lines) and materials subjected to the conditioning procedure (the solid lines) were investigated.

a

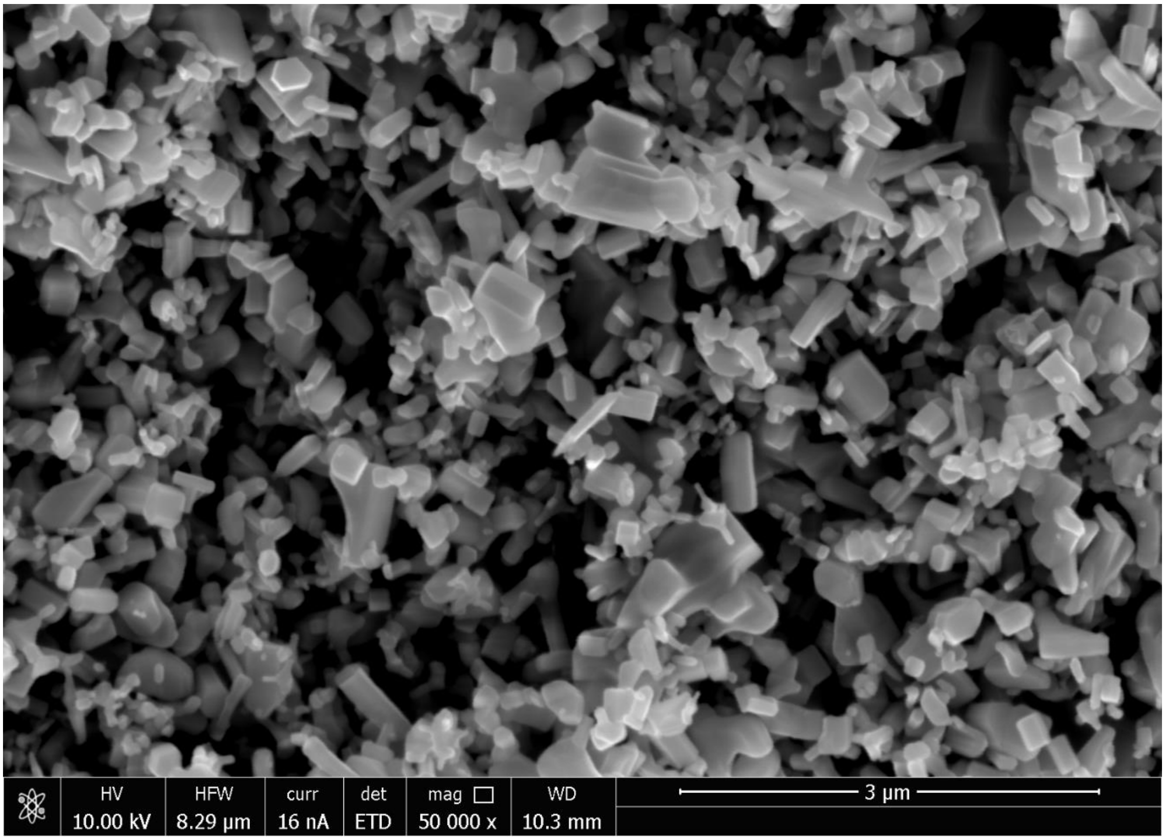
HV
10.00 kV
HFW
8.29 µm
curr
16 nA
det
ETD
mag
50 000 x
WD
10.3 mm
3 µm

b

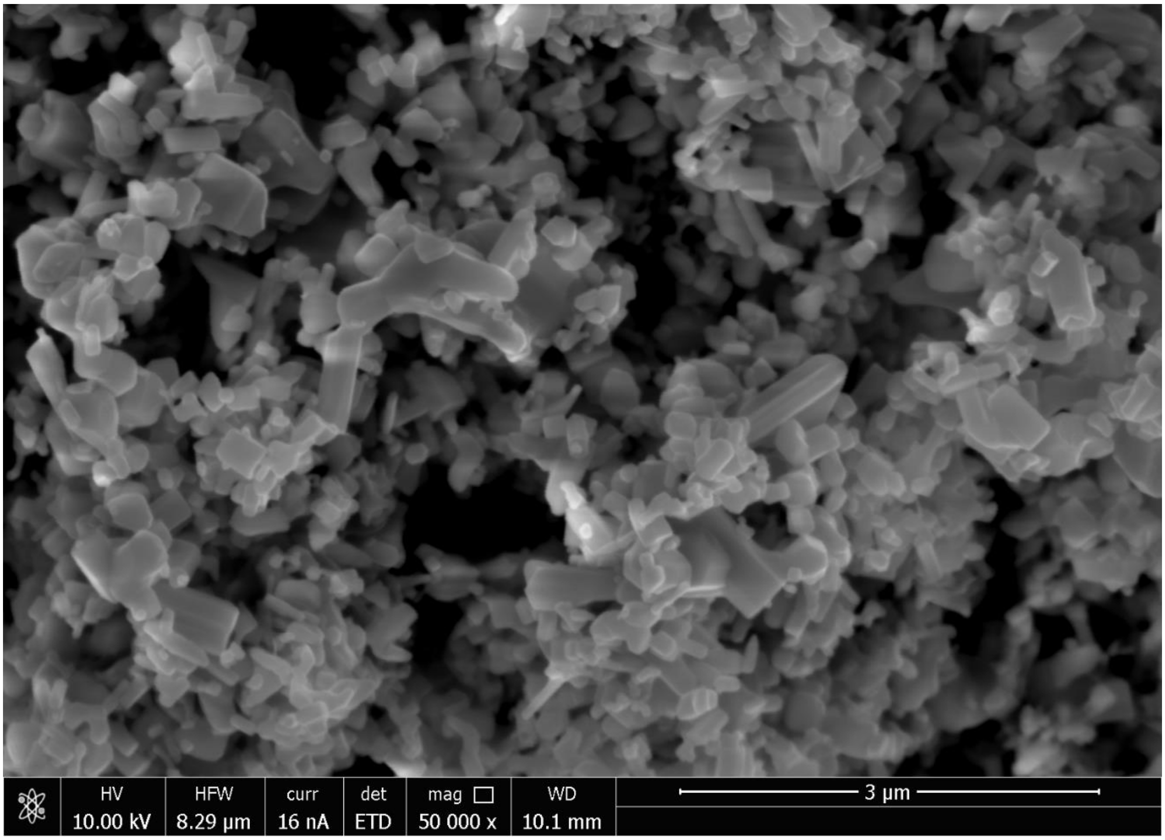
HV
10.00 kV
HFW
8.29 µm
curr
16 nA
det
ETD
mag
50 000 x
WD
10.1 mm
3 µm

c

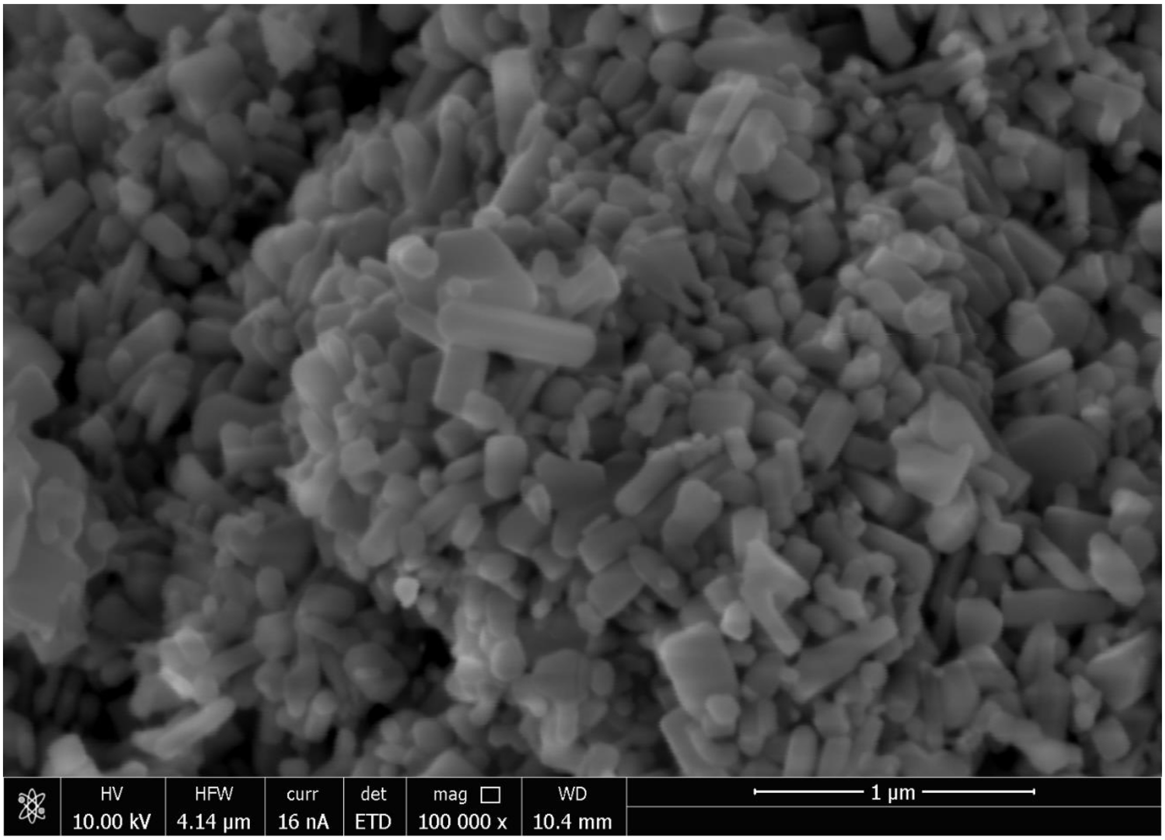
HV
10.00 kV
HFW
4.14 µm
curr
16 nA
det
ETD
mag
100 000 x
WD
10.4 mm
1 µm

d

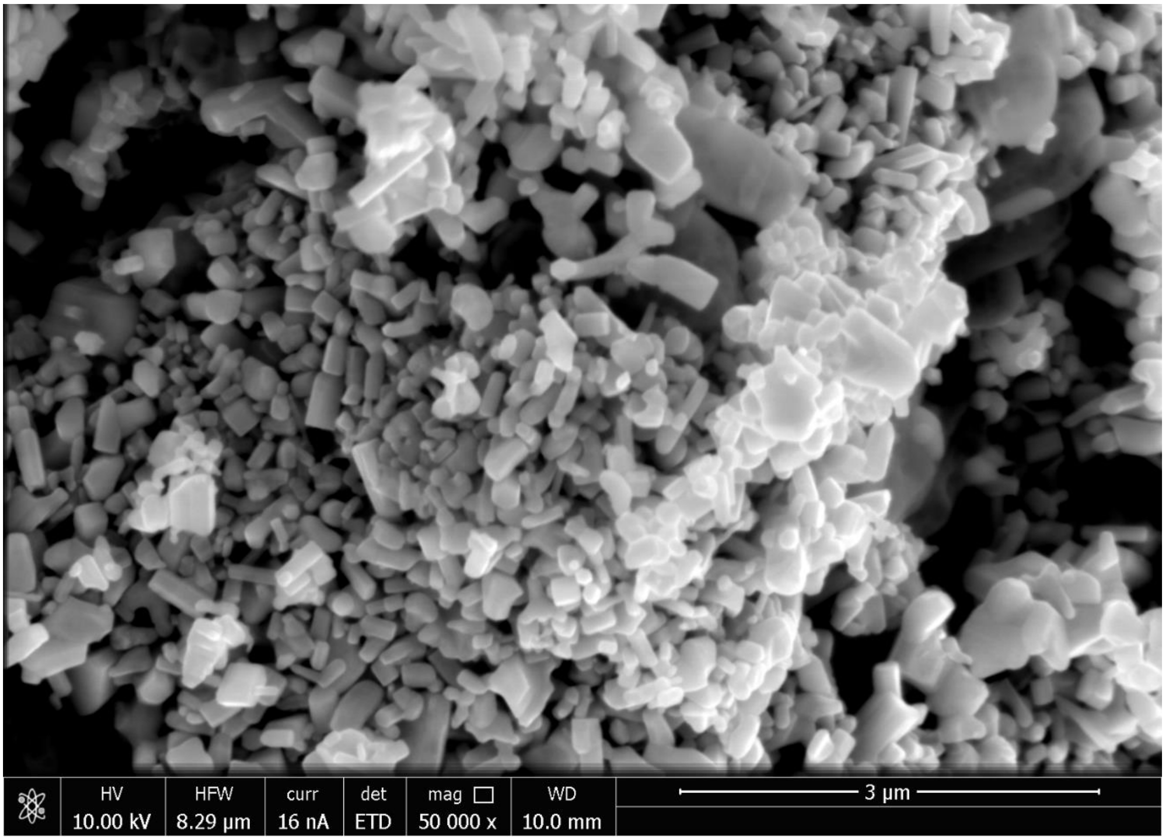
HV
10.00 kV
HFW
8.29 µm
curr
16 nA
det
ETD
mag
50 000 x
WD
10.0 mm
3 µm

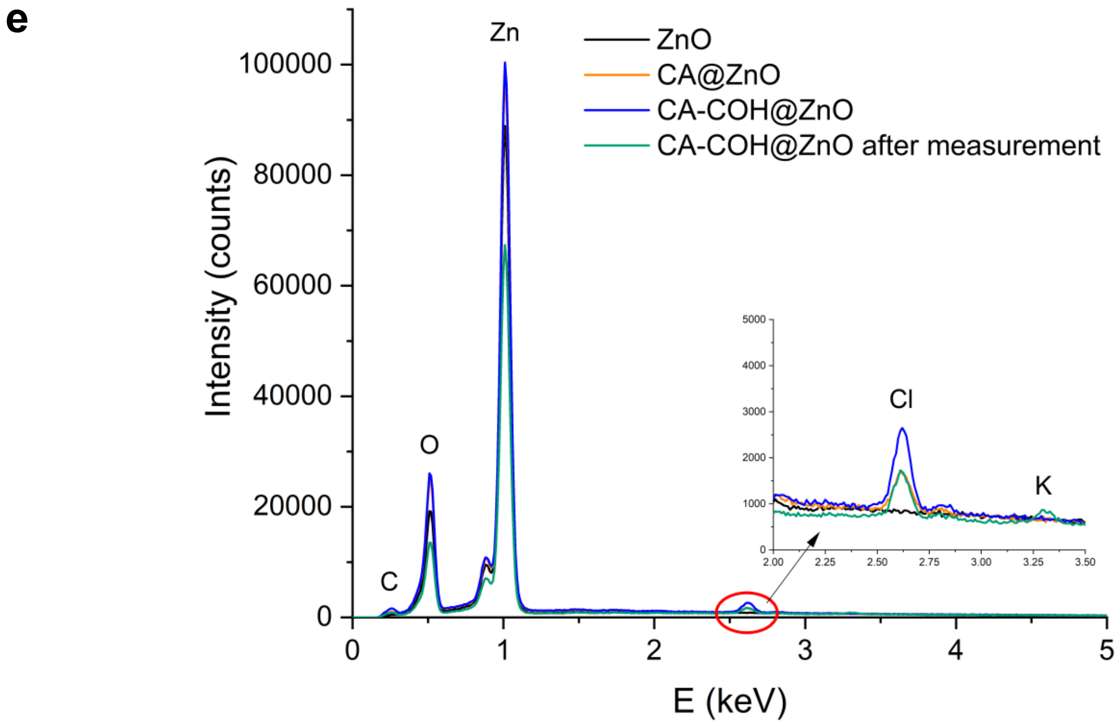

Supplementary Figure 17. Scanning electron micrographs and the EDS spectra of the investigated materials. Neat zinc oxide (a), CA@ZnO composite (b), CA-COH@ZnO fresh sample (c) and the ternary hybrid material after the conditioning procedure and LIIPS effect measurements (d) were examined. No significant differences in the morphology between considered systems have been observed (although the ternary material seems to exhibit a slightly higher tendency towards agglomeration). The EDS spectra (e) include an additional Cl signal for samples containing chloranilic acid and for the material subjected to measurements in KNO_3 solution a weak peak attributed to potassium was observed.
