# Supplementary figures and images for: Light intensity-induced photocurrent switching effect
Source: Nat Commun. 2020 Feb 12;11:854. doi: 10.1038/s41467-020-14675-5 (PMC7016128; doi:10.1038/s41467-020-14675-5)

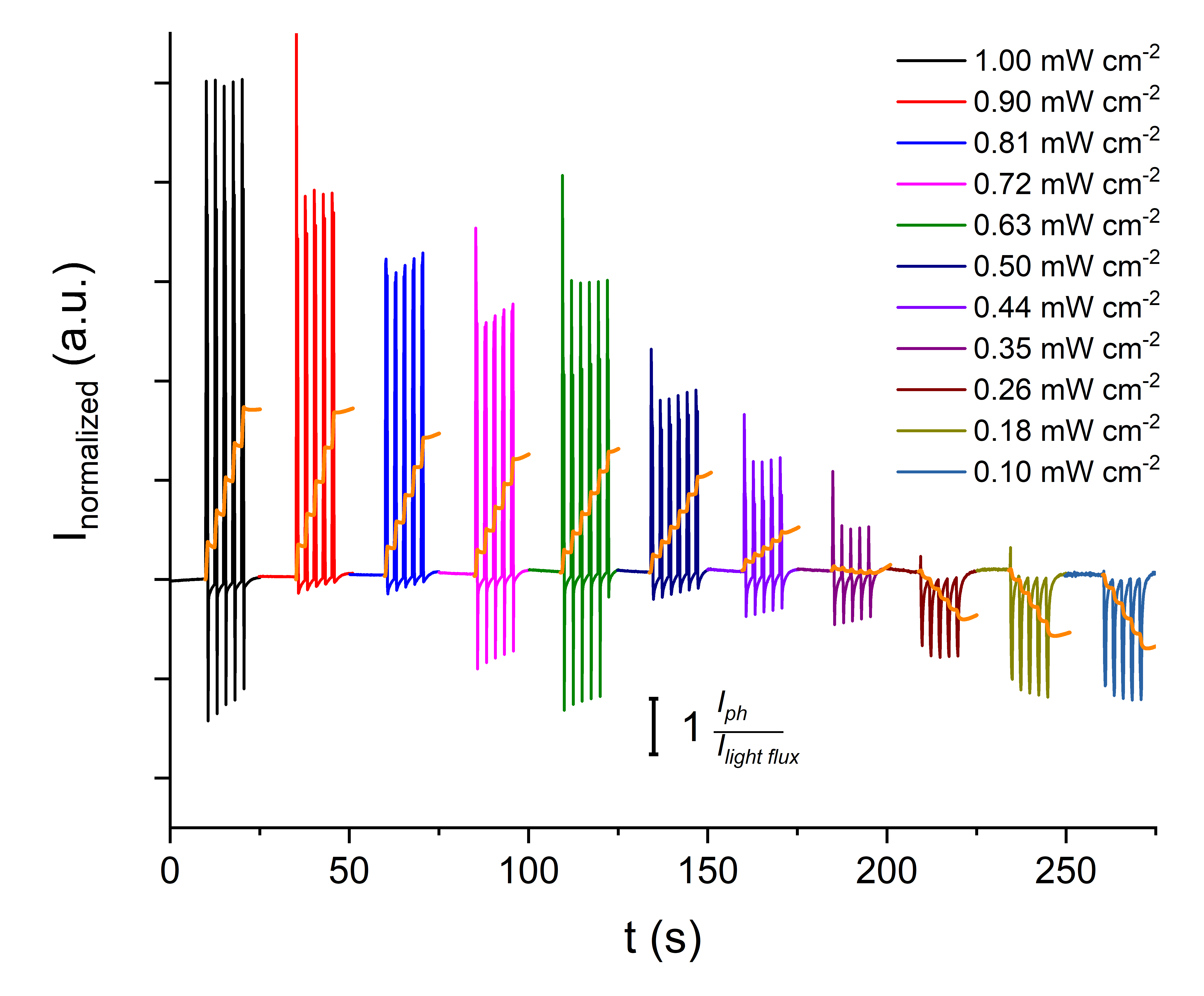

Supplement: Supplementary file 3 — Figure S1 to S17 [file 41467_2020_14675_MOESM3_ESM.zip › Figure S1.png]

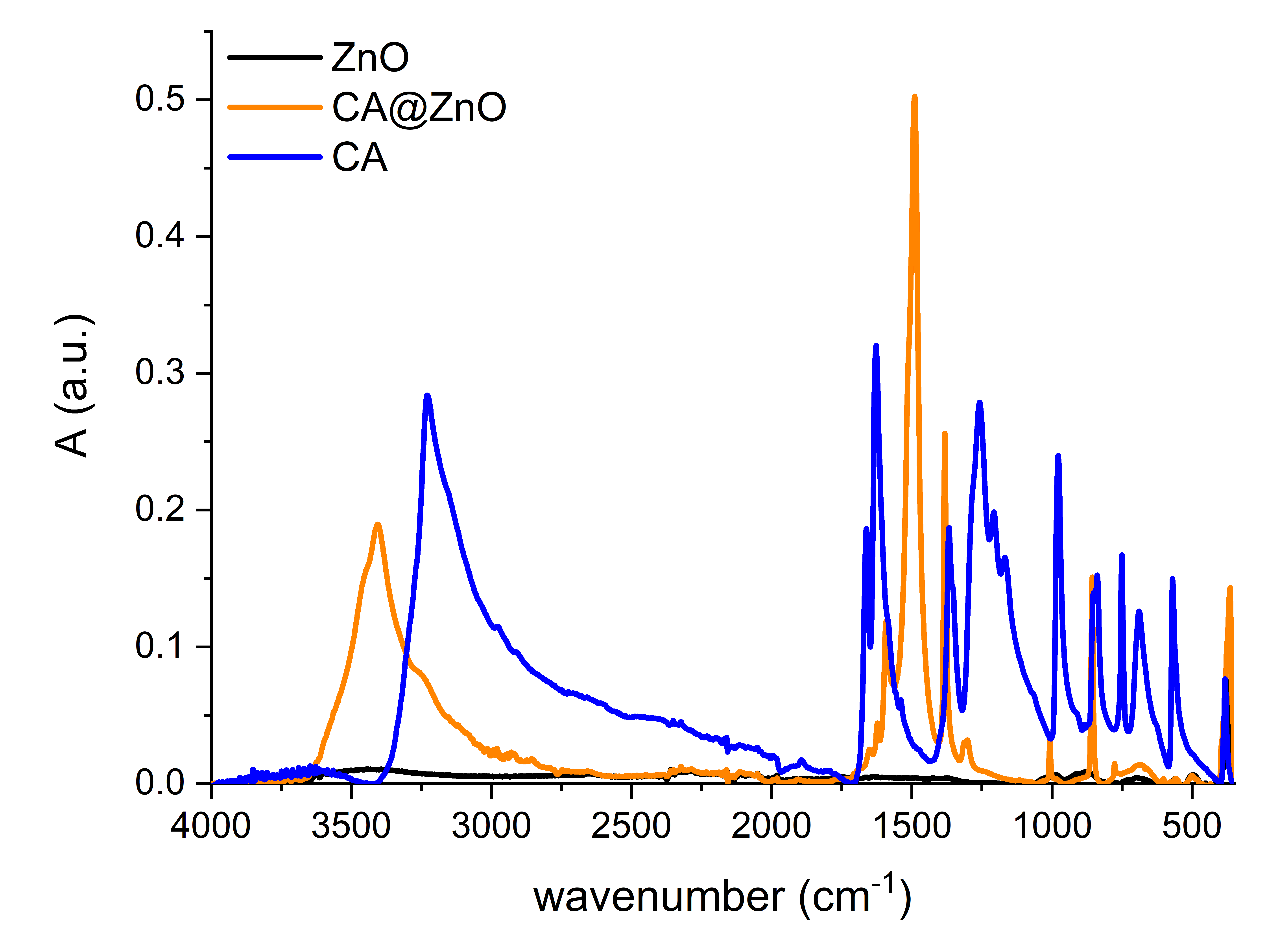

Supplement: Supplementary file 3 — Figure S1 to S17 [file 41467_2020_14675_MOESM3_ESM.zip › Figure S10.png]

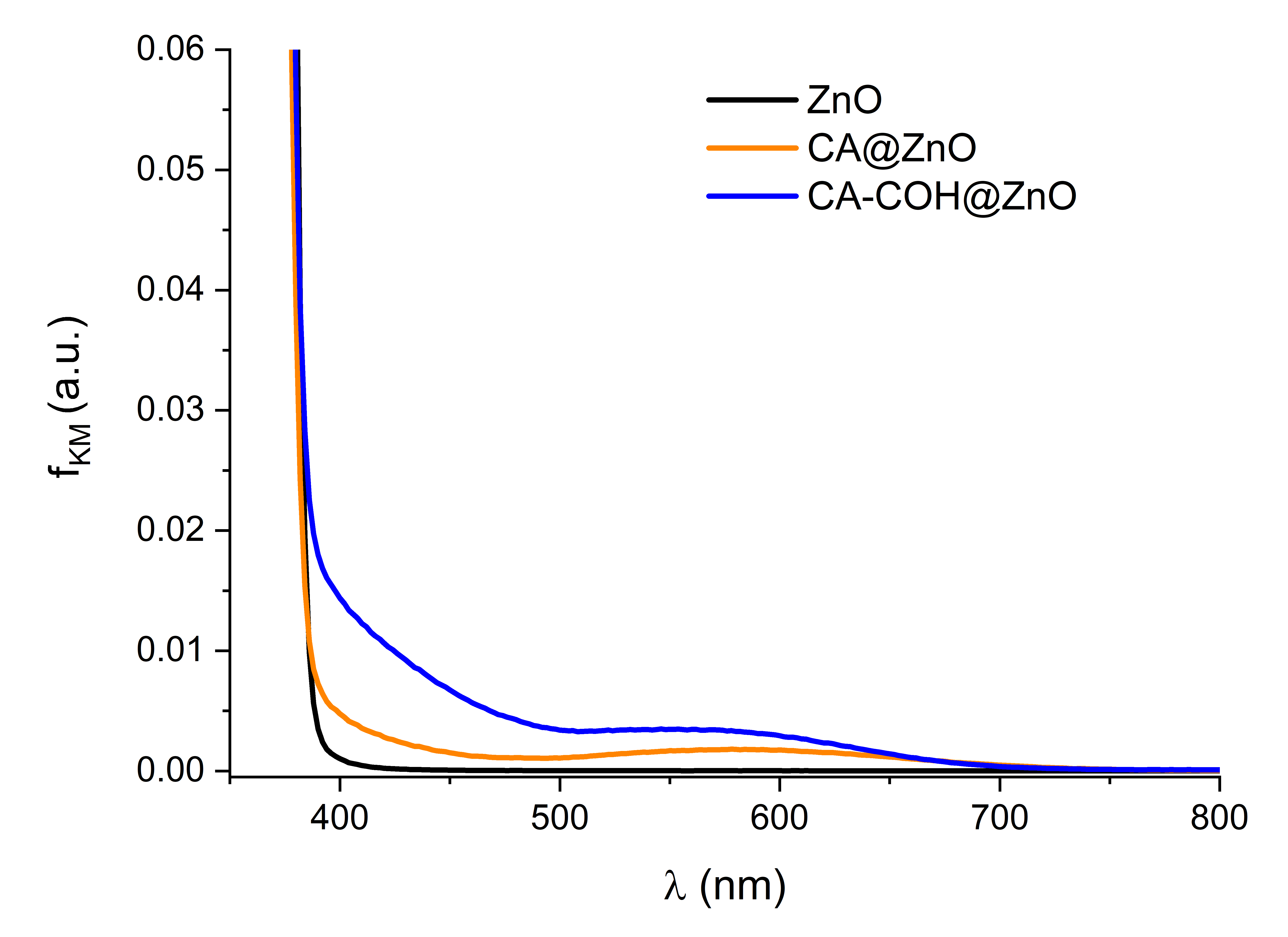

Supplement: Supplementary file 3 — Figure S1 to S17 [file 41467_2020_14675_MOESM3_ESM.zip › Figure S11.png]

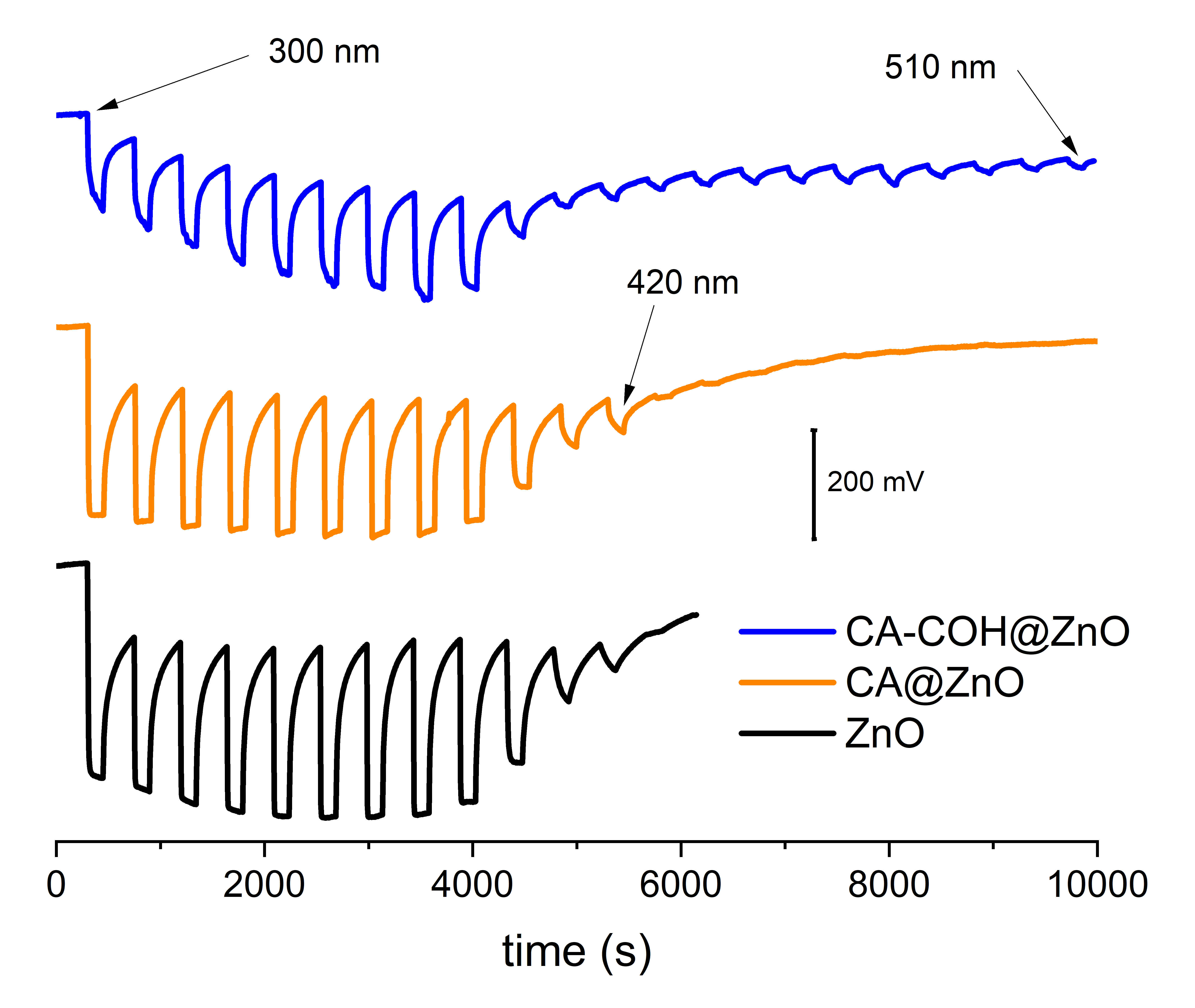

Supplement: Supplementary file 3 — Figure S1 to S17 [file 41467_2020_14675_MOESM3_ESM.zip › Figure S12.png]

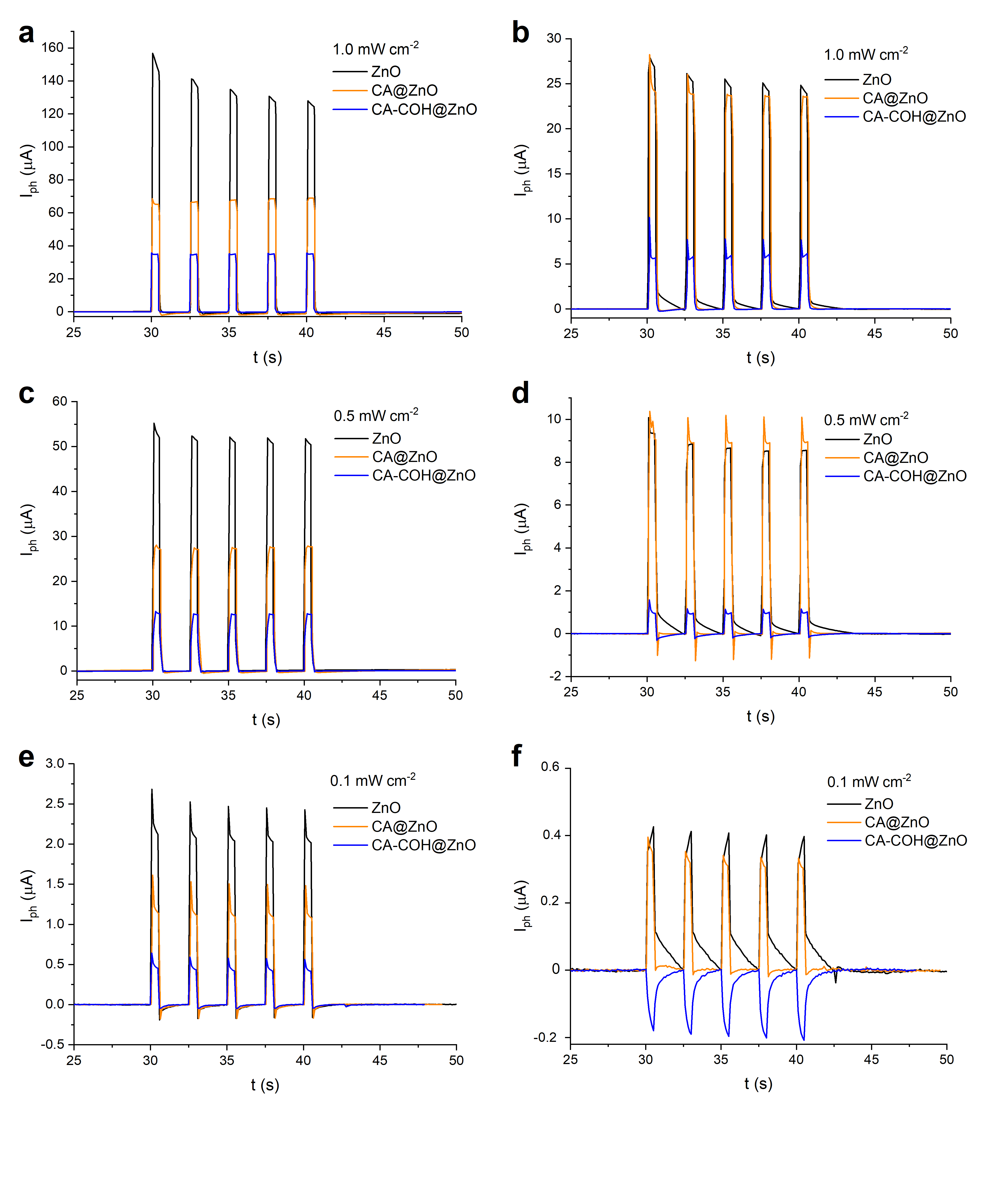

Supplement: Supplementary file 3 — Figure S1 to S17 [file 41467_2020_14675_MOESM3_ESM.zip › Figure S13.png]

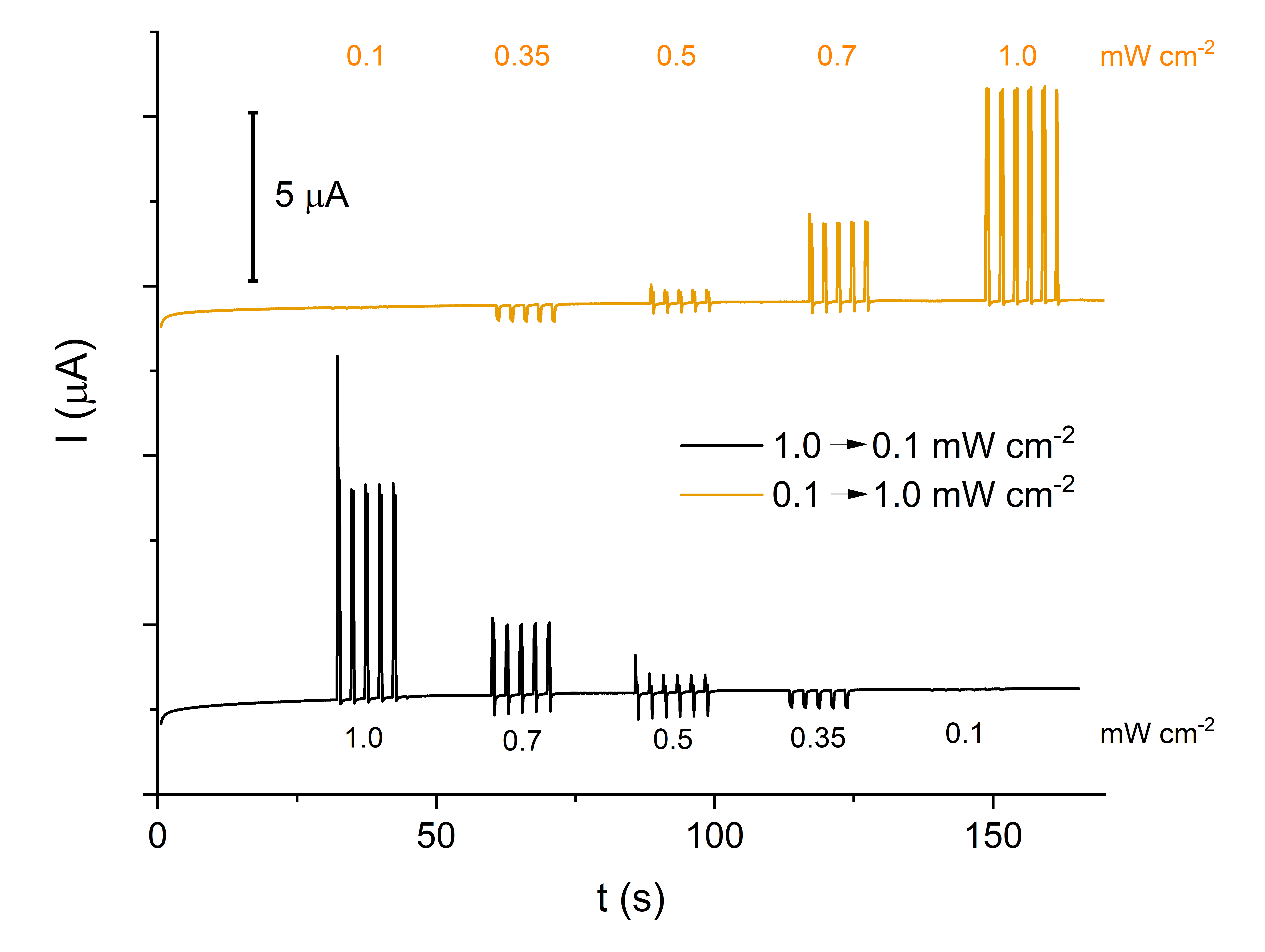

Supplement: Supplementary file 3 — Figure S1 to S17 [file 41467_2020_14675_MOESM3_ESM.zip › Figure S14.png]

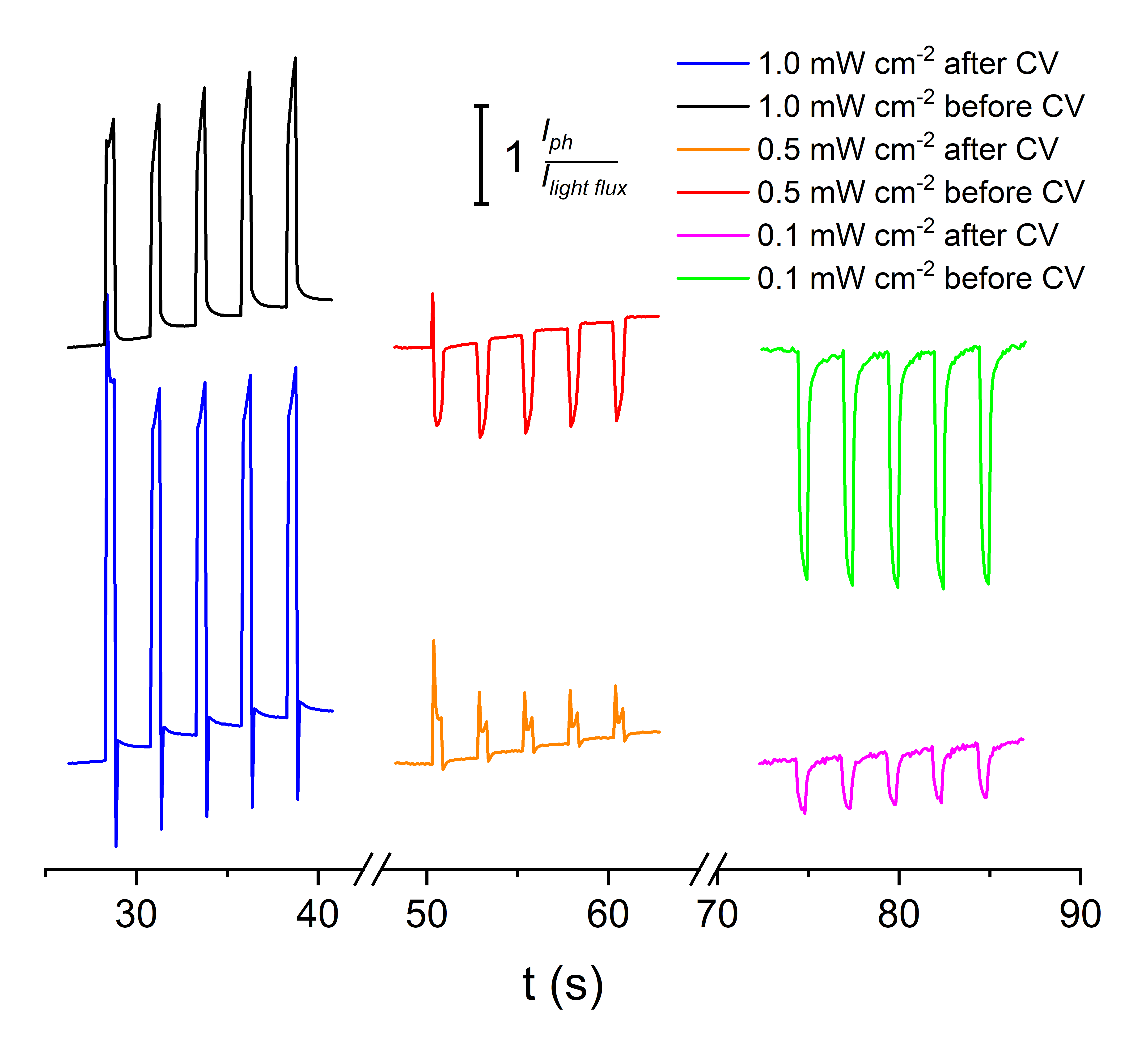

Supplement: Supplementary file 3 — Figure S1 to S17 [file 41467_2020_14675_MOESM3_ESM.zip › Figure S15.png]

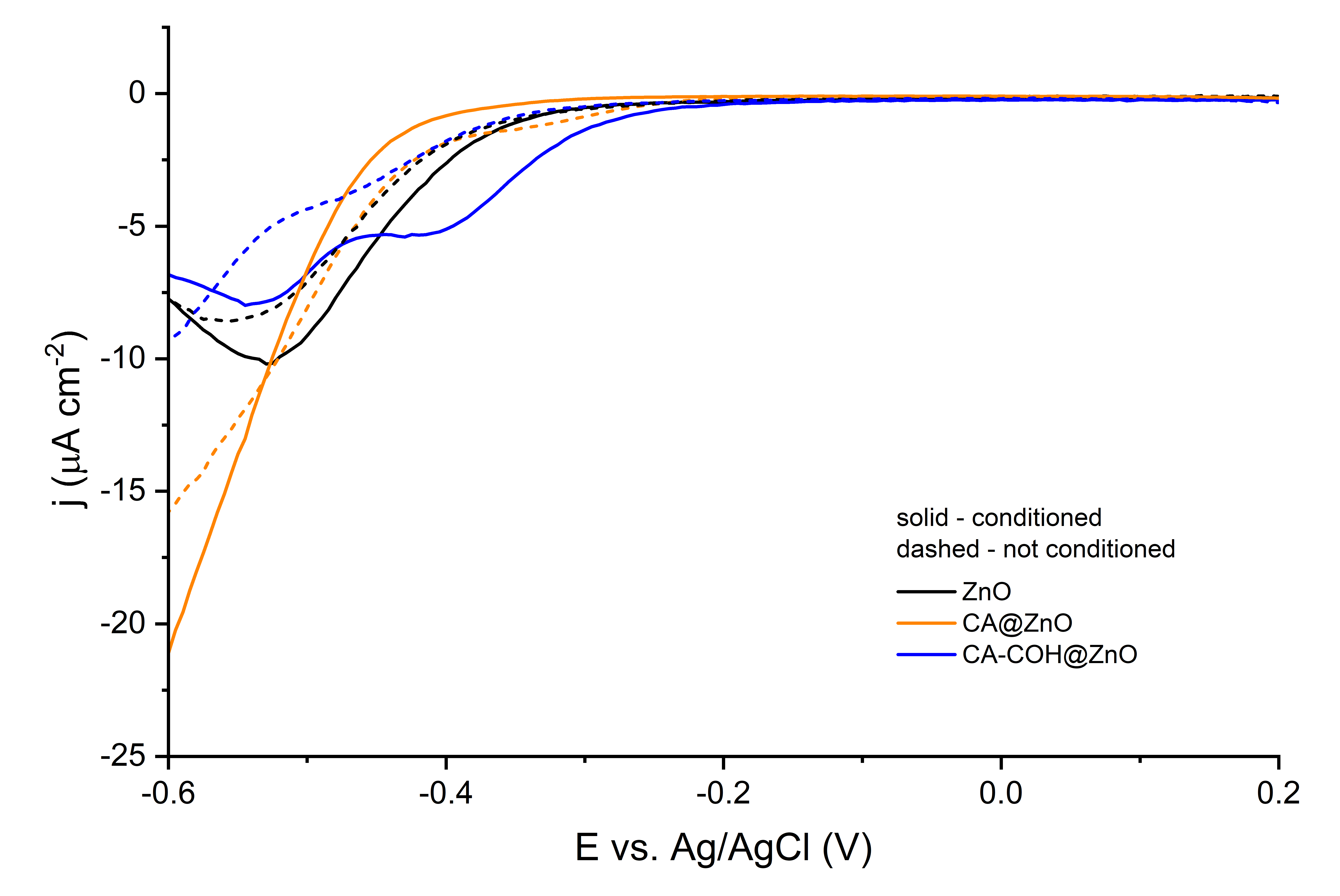

Supplement: Supplementary file 3 — Figure S1 to S17 [file 41467_2020_14675_MOESM3_ESM.zip › Figure S16.png]

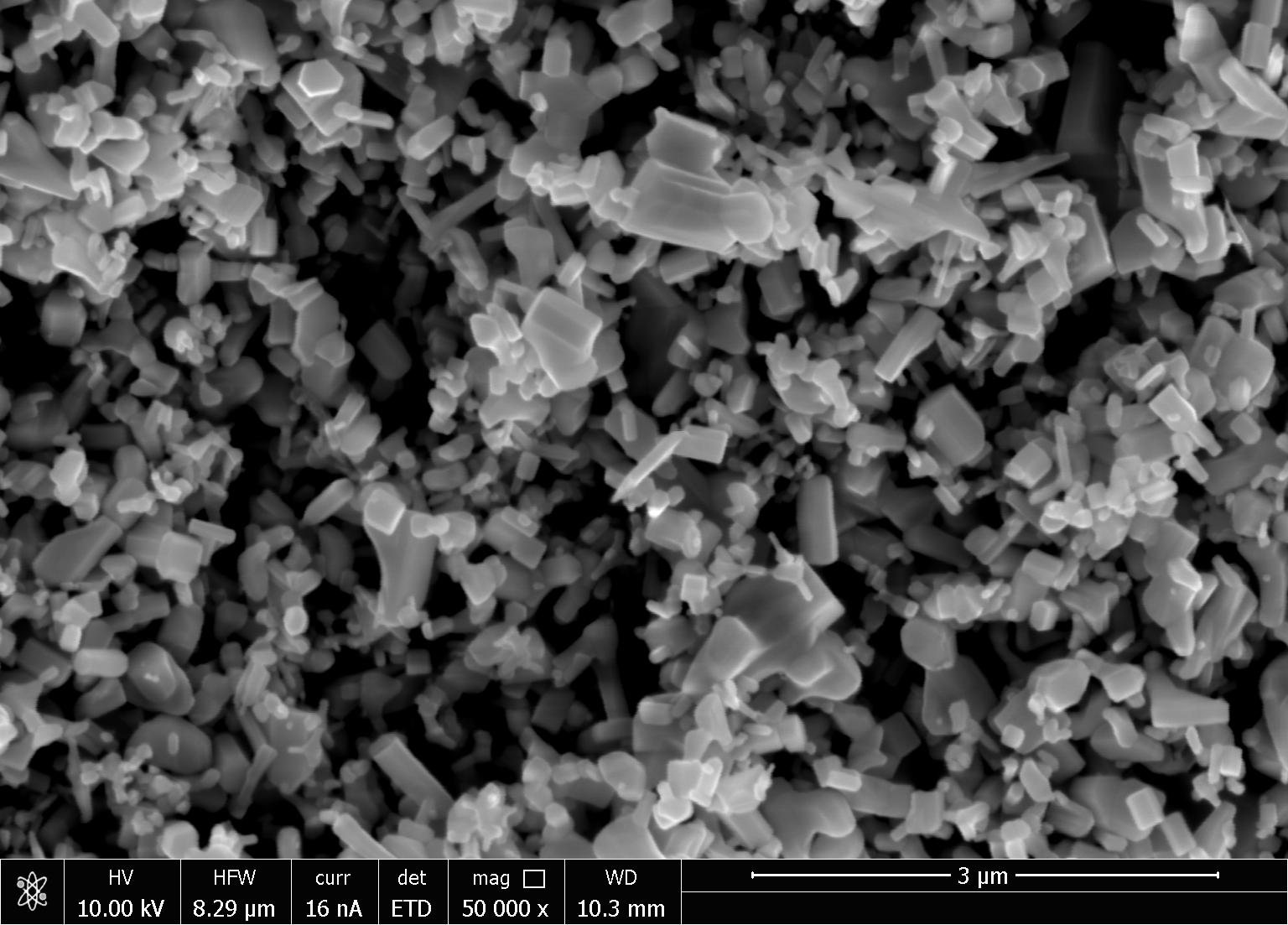

Supplement: Supplementary file 3 — Figure S1 to S17 [file 41467_2020_14675_MOESM3_ESM.zip › Figure S17a.tif]

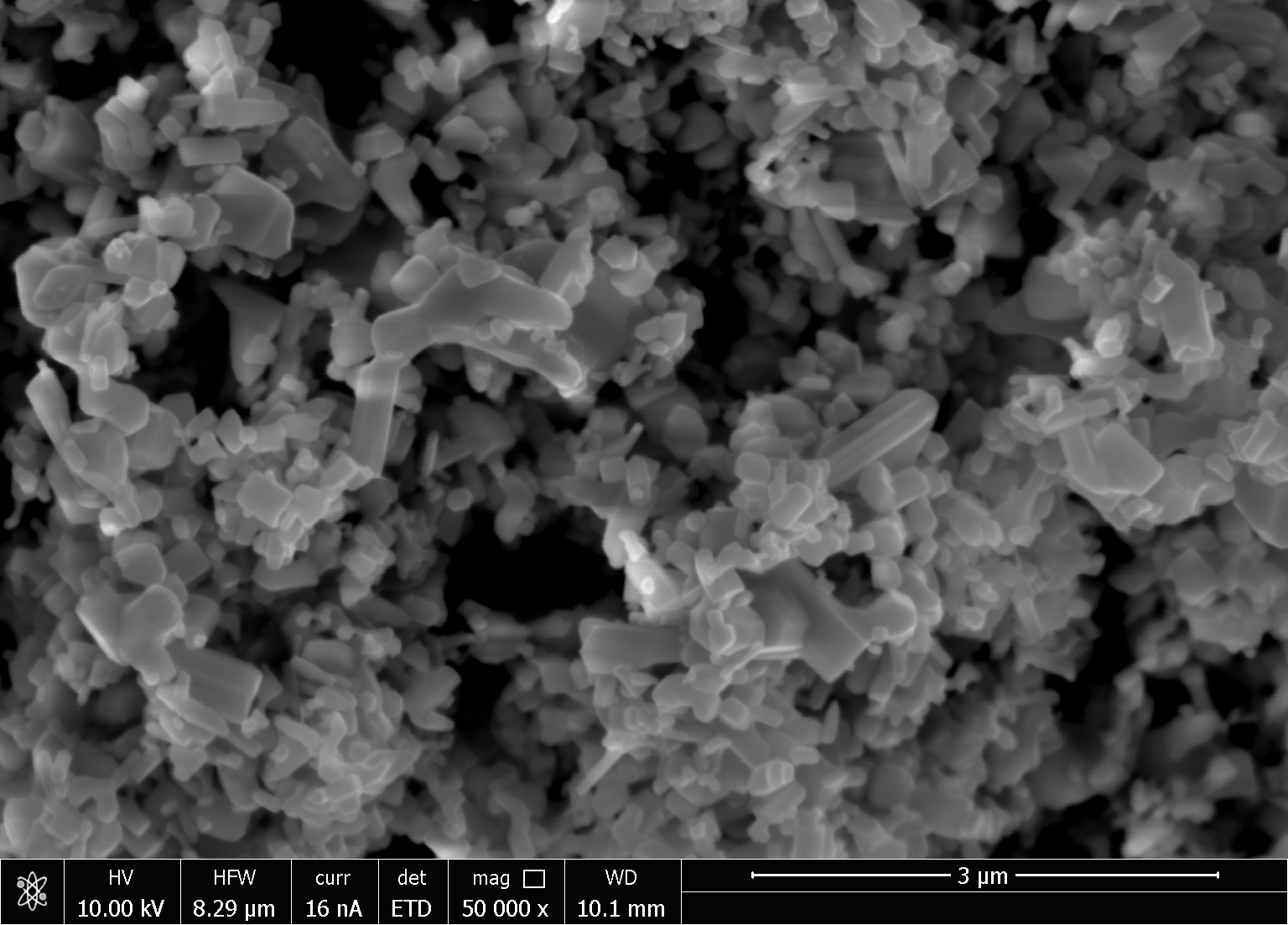

Supplement: Supplementary file 3 — Figure S1 to S17 [file 41467_2020_14675_MOESM3_ESM.zip › Figure S17b.tif]

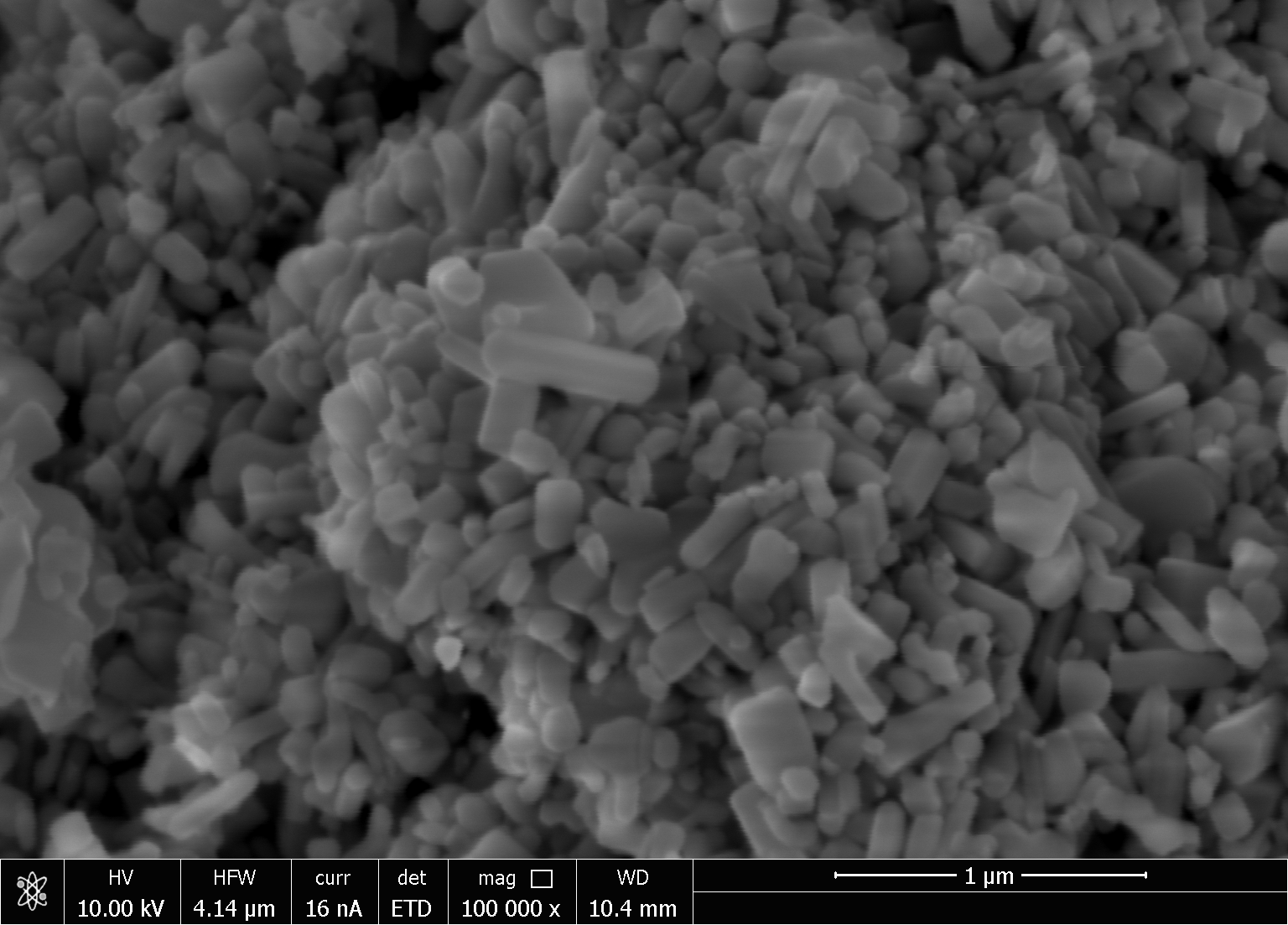

Supplement: Supplementary file 3 — Figure S1 to S17 [file 41467_2020_14675_MOESM3_ESM.zip › Figure S17c.tif]

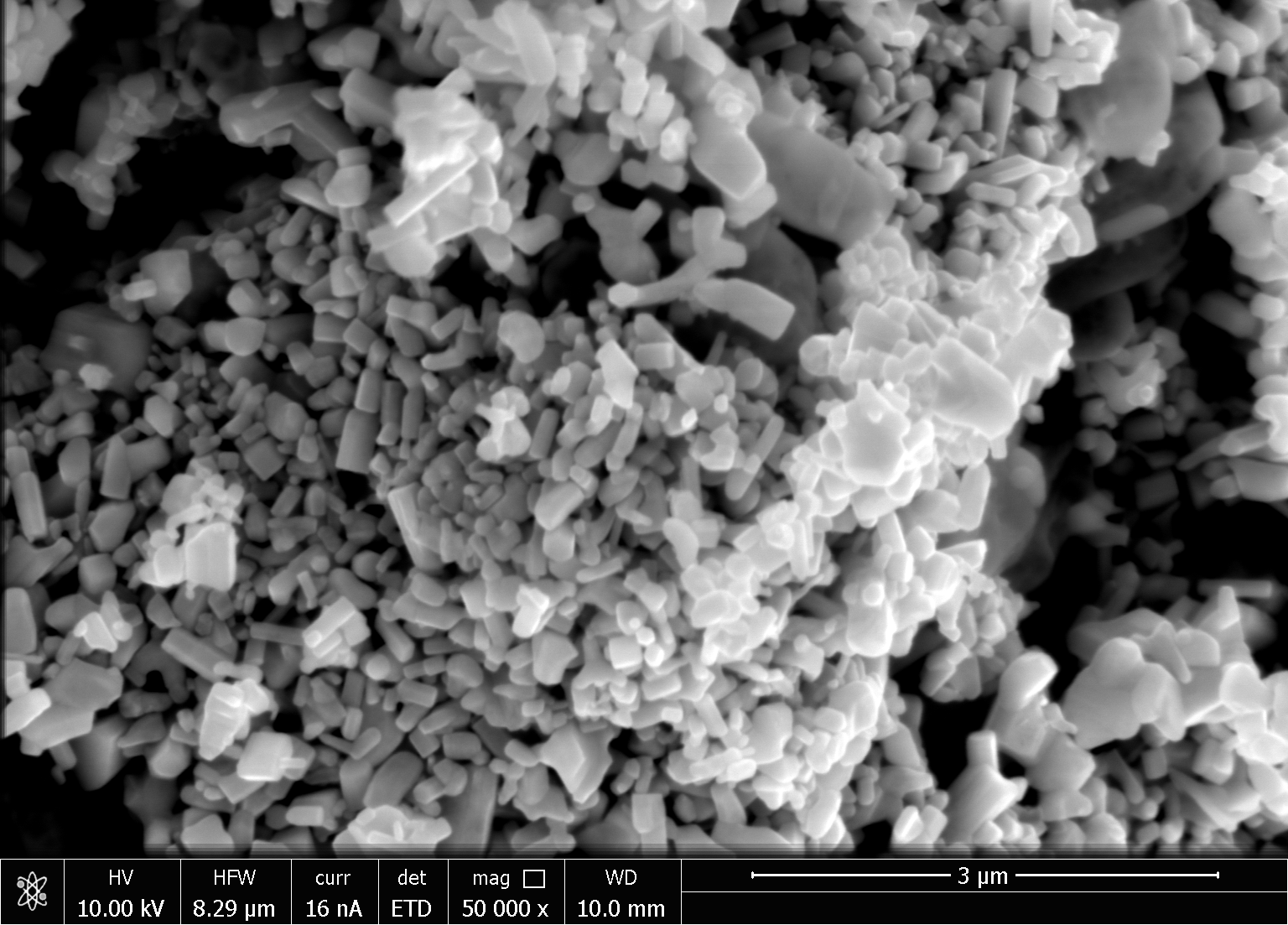

Supplement: Supplementary file 3 — Figure S1 to S17 [file 41467_2020_14675_MOESM3_ESM.zip › Figure S17d.tif]

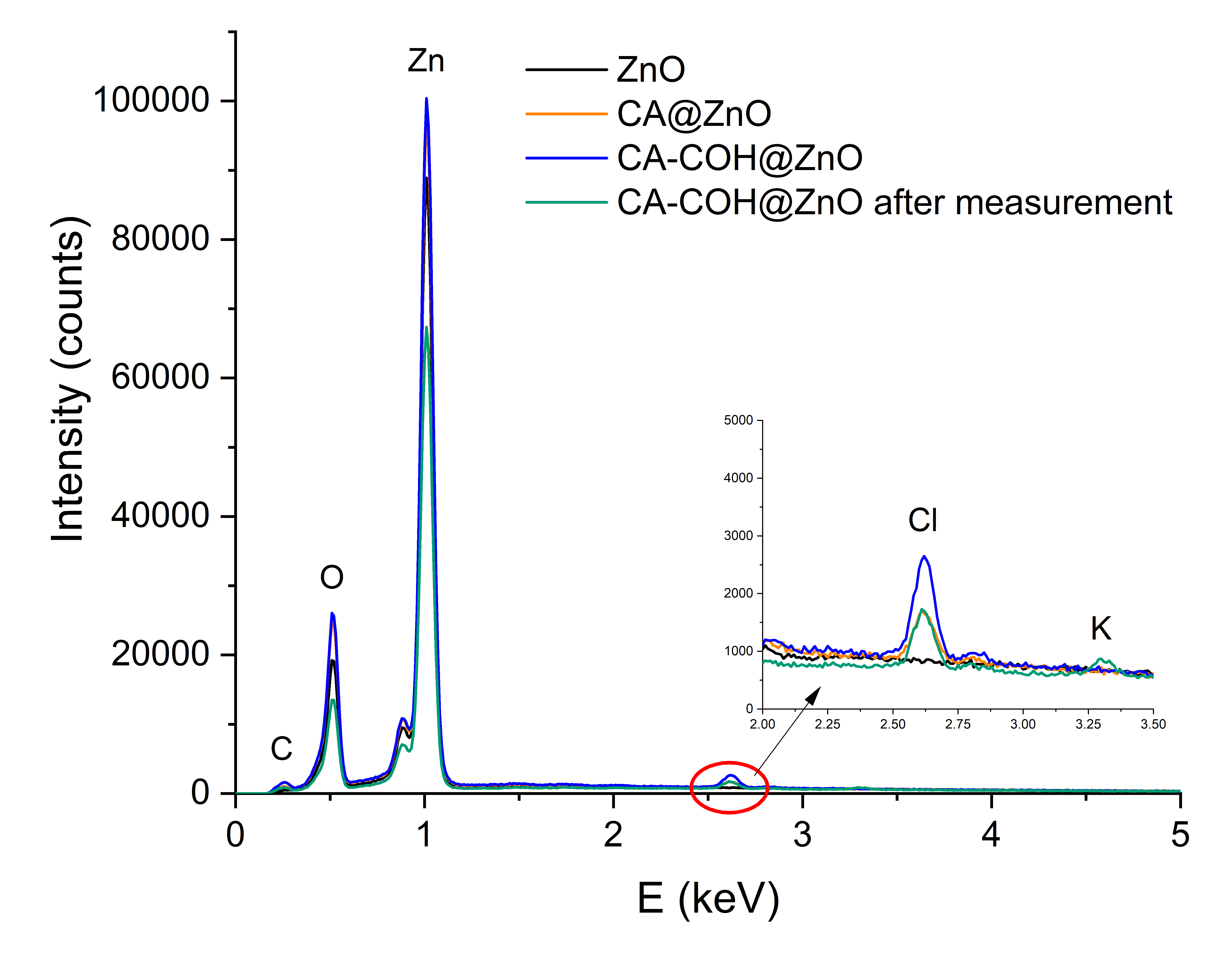

Supplement: Supplementary file 3 — Figure S1 to S17 [file 41467_2020_14675_MOESM3_ESM.zip › Figure S17e.png]

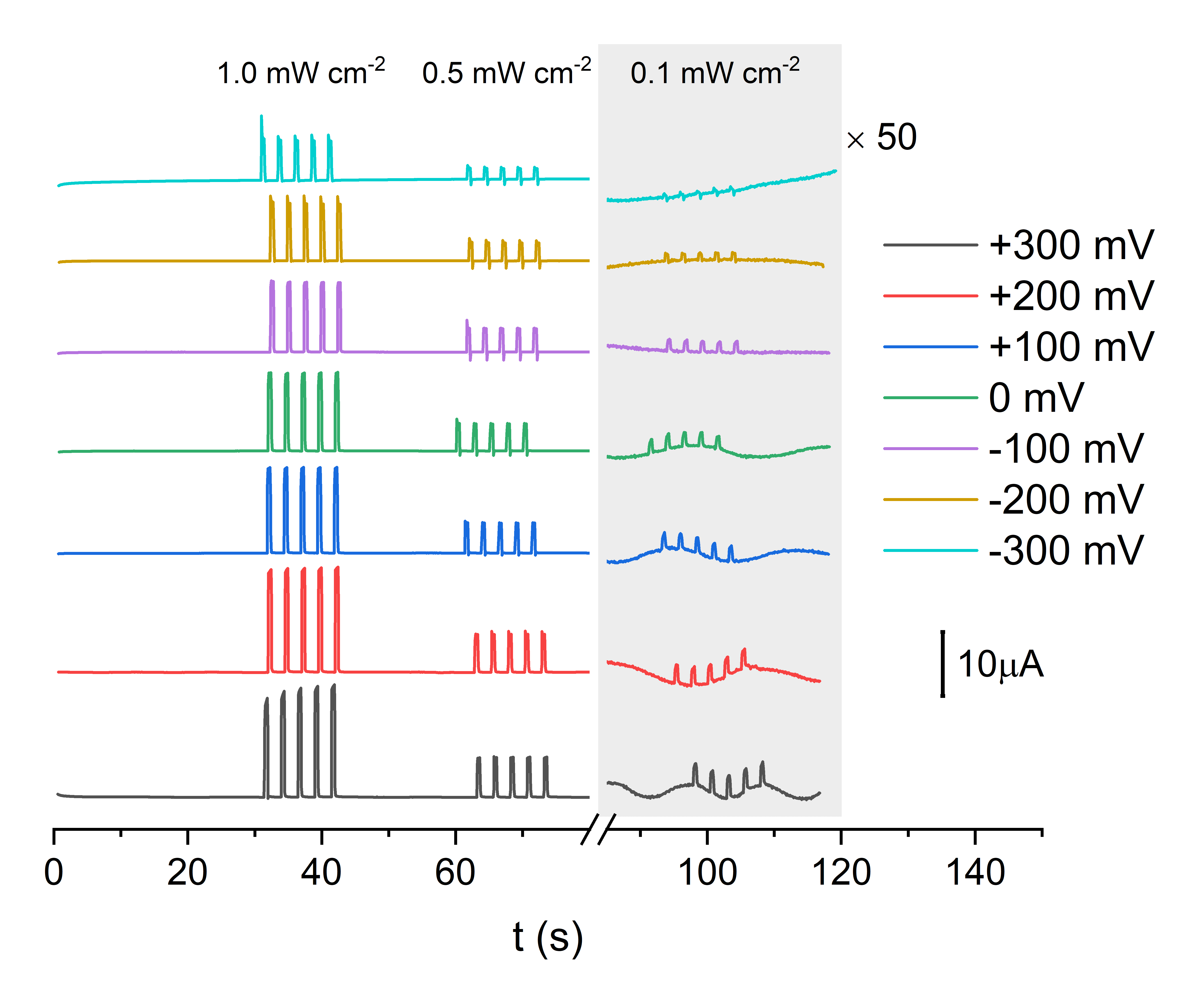

Supplement: Supplementary file 3 — Figure S1 to S17 [file 41467_2020_14675_MOESM3_ESM.zip › Figure S2.png]

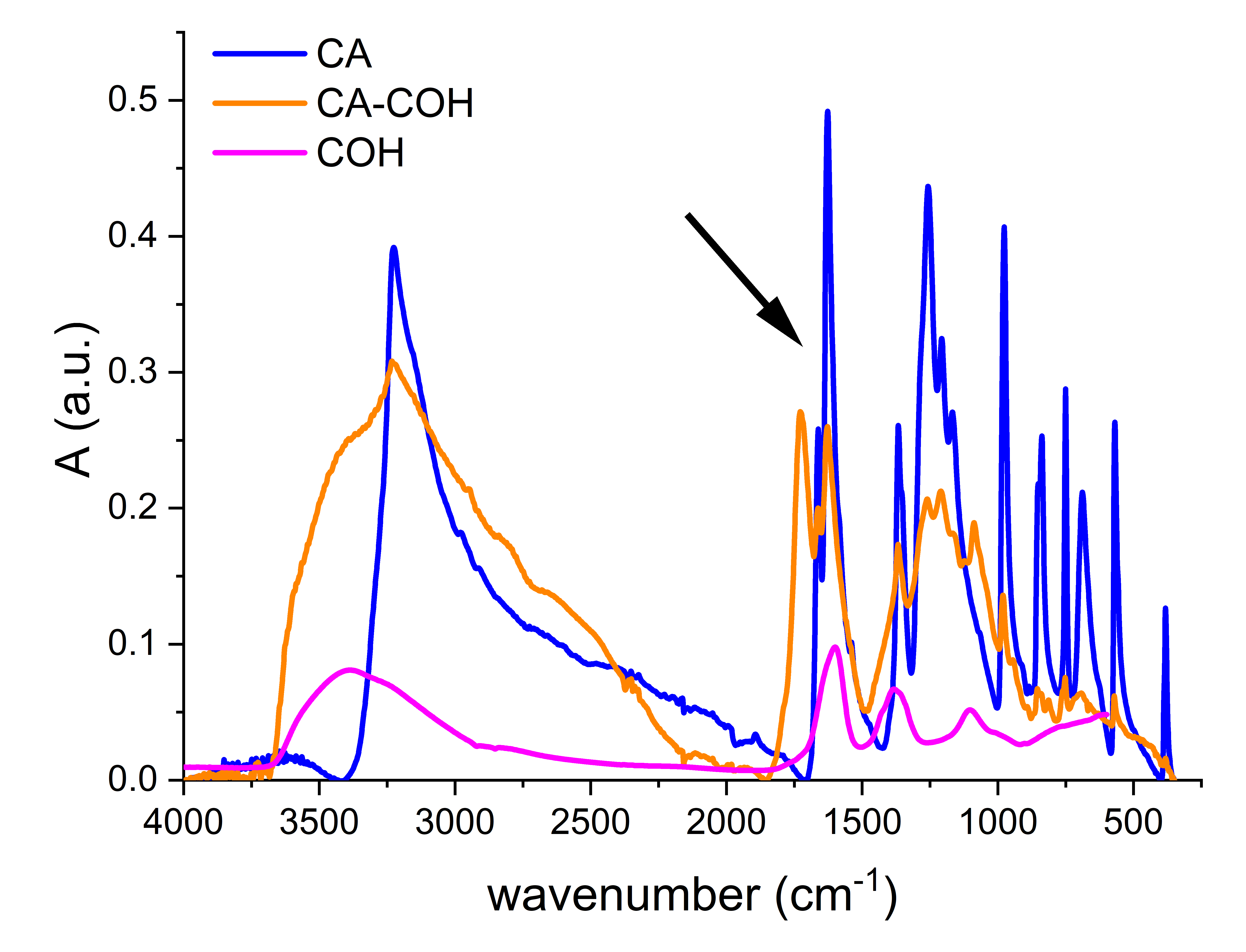

Supplement: Supplementary file 3 — Figure S1 to S17 [file 41467_2020_14675_MOESM3_ESM.zip › Figure S3.png]

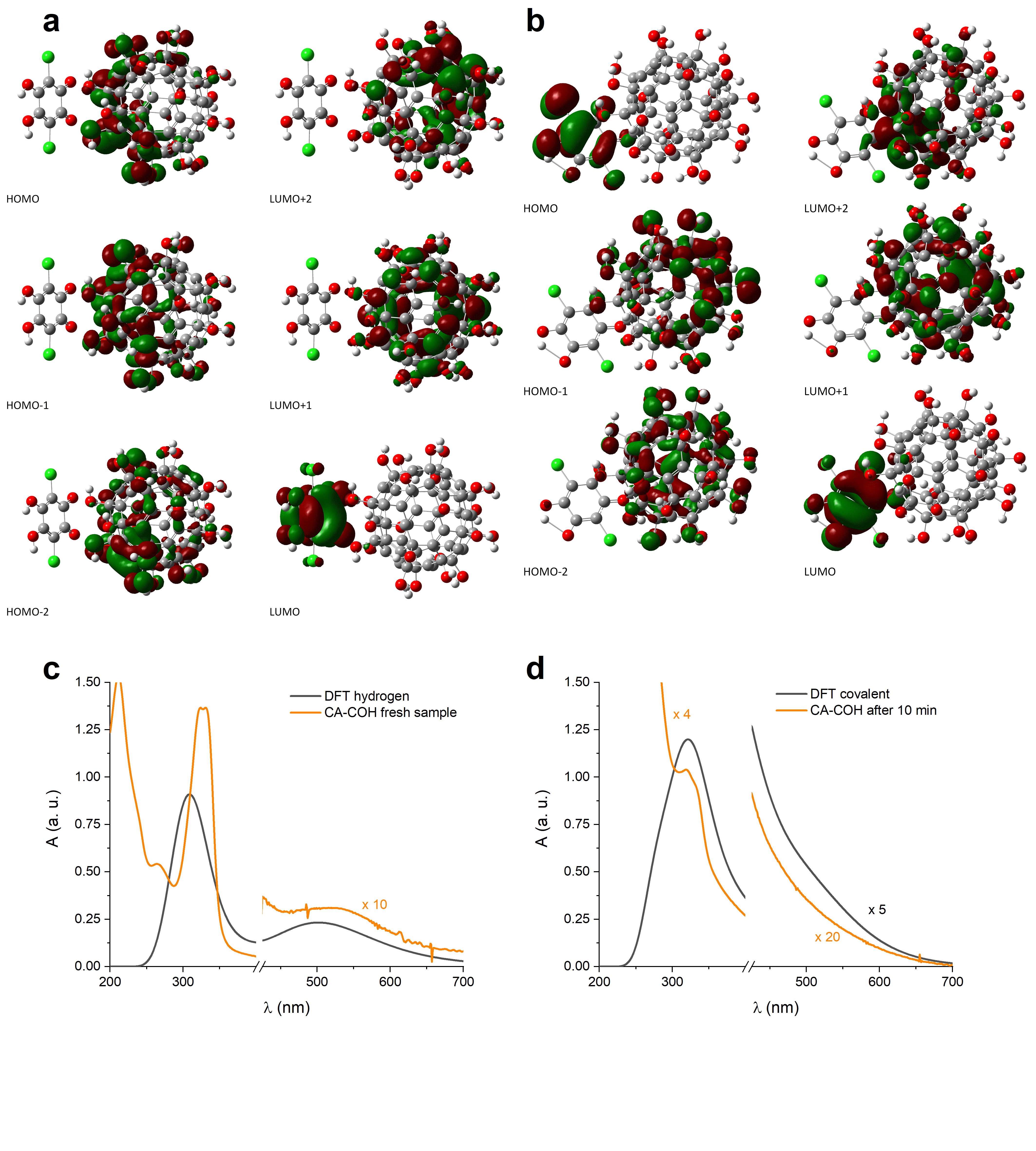

Supplement: Supplementary file 3 — Figure S1 to S17 [file 41467_2020_14675_MOESM3_ESM.zip › Figure S4.png]

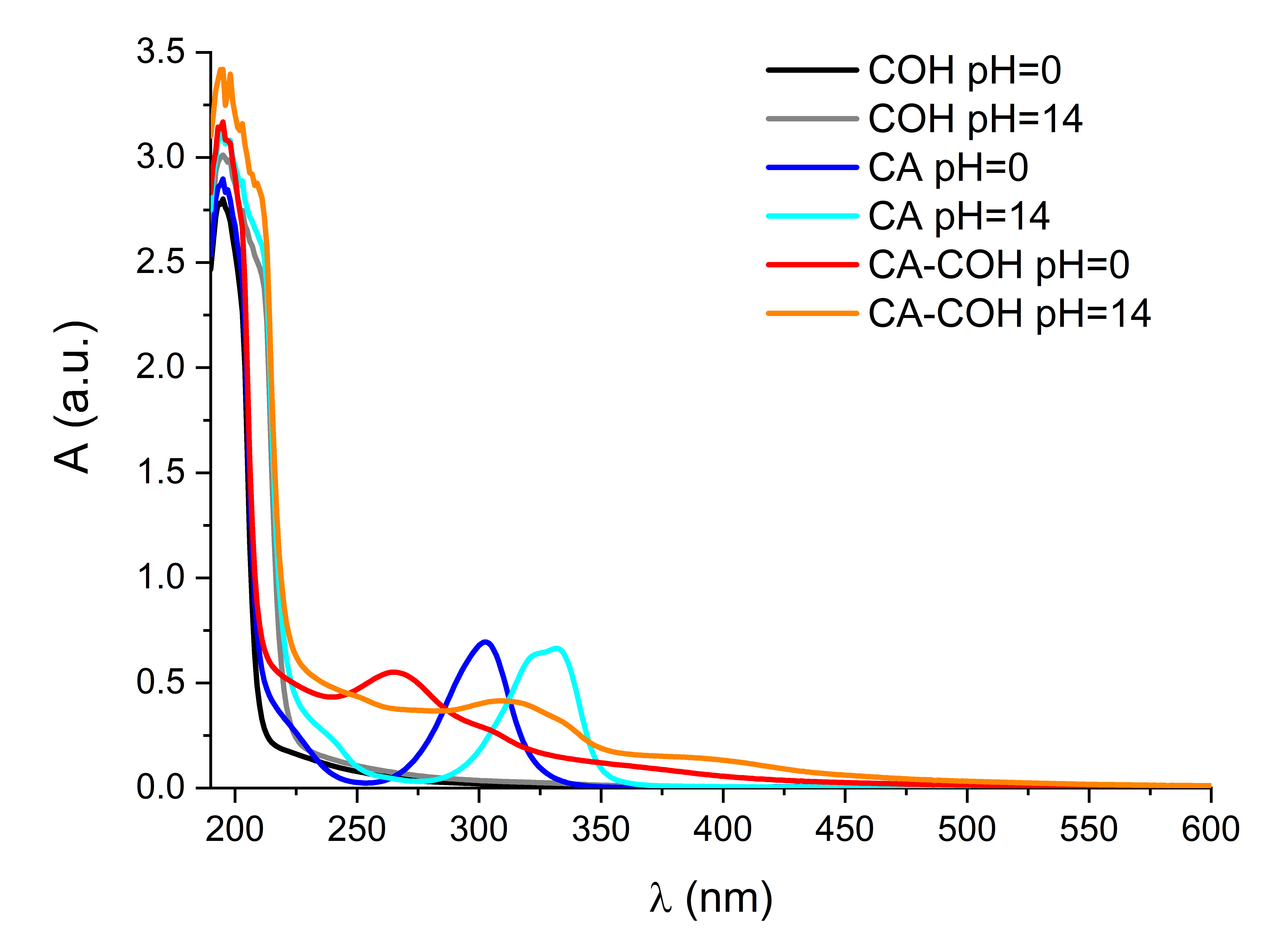

Supplement: Supplementary file 3 — Figure S1 to S17 [file 41467_2020_14675_MOESM3_ESM.zip › Figure S5.png]

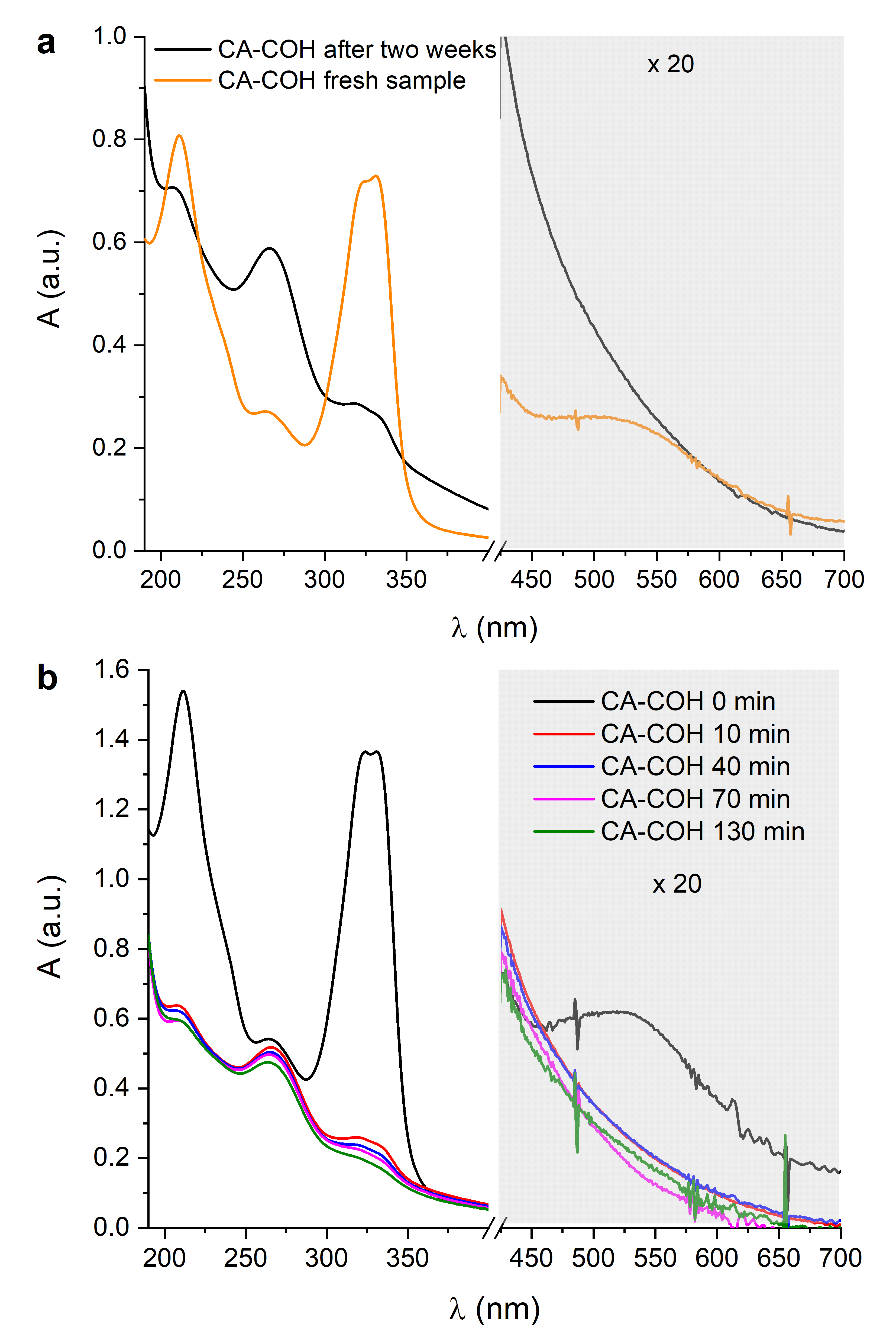

Supplement: Supplementary file 3 — Figure S1 to S17 [file 41467_2020_14675_MOESM3_ESM.zip › Figure S6.png]

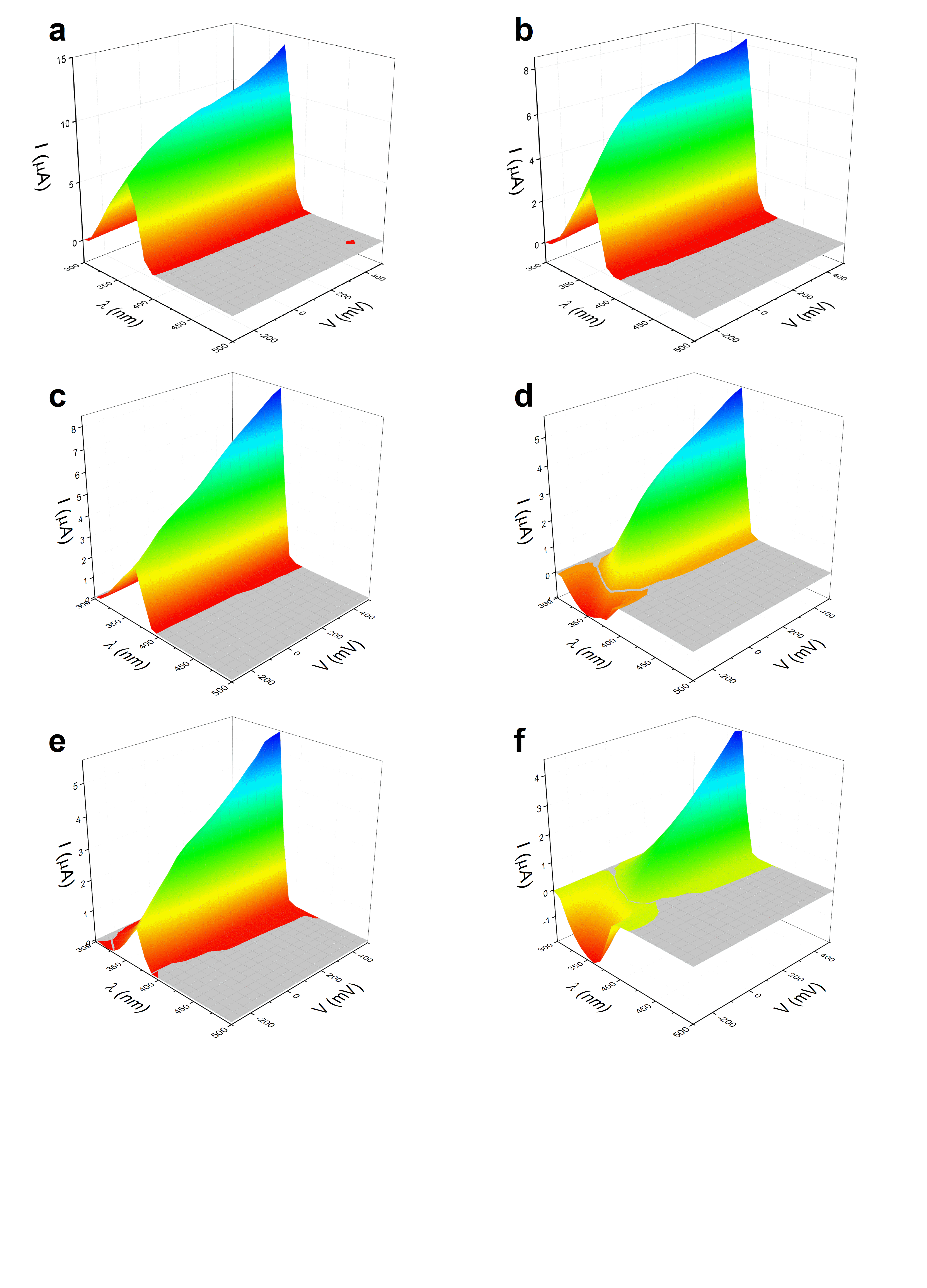

Supplement: Supplementary file 3 — Figure S1 to S17 [file 41467_2020_14675_MOESM3_ESM.zip › Figure S7.png]

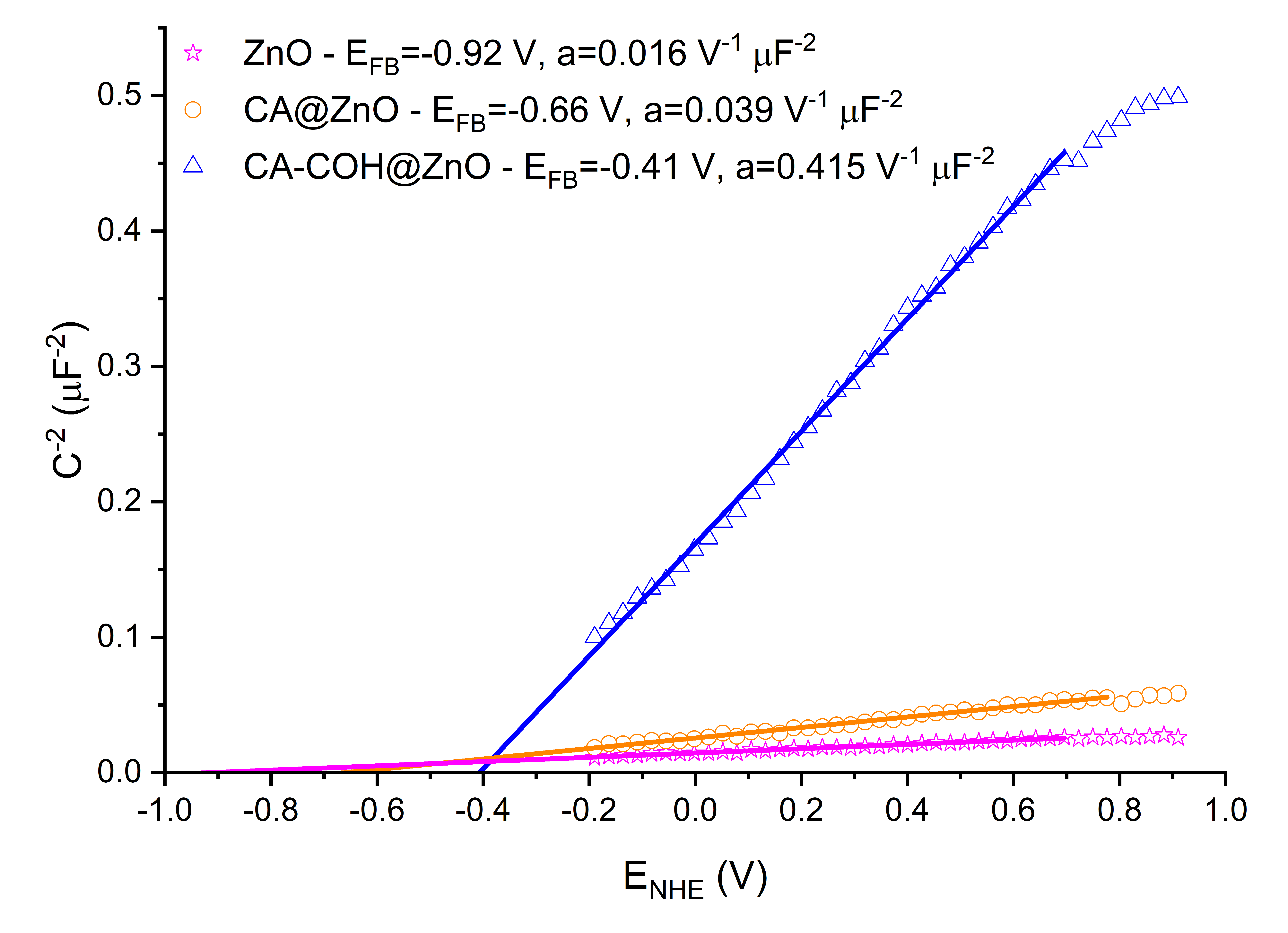

Supplement: Supplementary file 3 — Figure S1 to S17 [file 41467_2020_14675_MOESM3_ESM.zip › Figure S8.png]

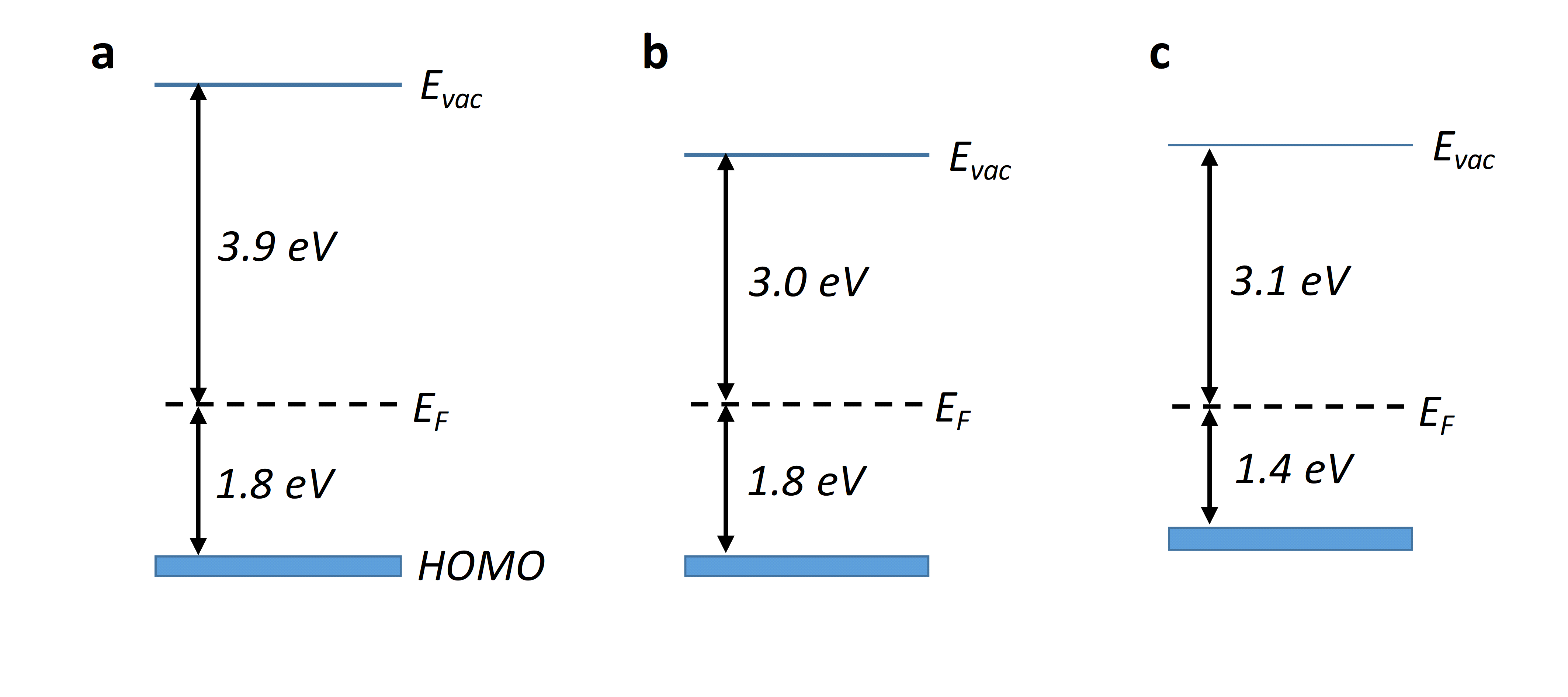

Supplement: Supplementary file 3 — Figure S1 to S17 [file 41467_2020_14675_MOESM3_ESM.zip › Figure S9.png]

## Slide 1
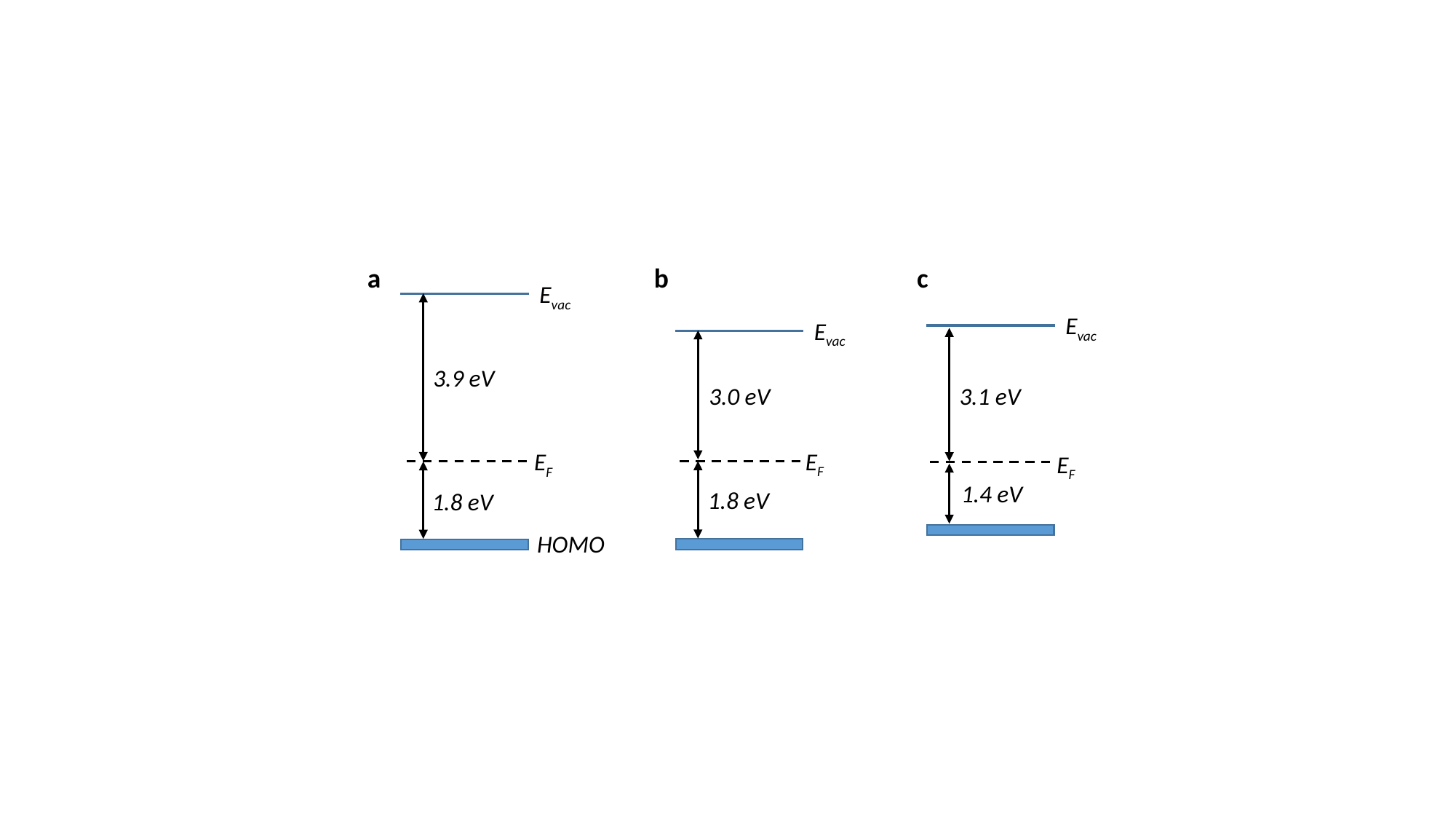

a
b
c
Evac
Evac
Evac
3.9 eV
3.1 eV
3.0 eV
EF
EF
EF
1.4 eV
1.8 eV
1.8 eV
HOMO

Supplement: Supplementary file 3 — Figure S1 to S17 [file 41467_2020_14675_MOESM3_ESM.zip › Figure S9.pptx]
